# Non-Technical Competences (Soft Skills) in Dentistry: Patients’ Perceptions in Private Dental Practices in Iași, Romania

**DOI:** 10.3390/healthcare14182906

**Published:** 2026-09-08

**Authors:** Magda-Ecaterina Antohe, Monica Silvia Tatarciuc, Cristina Gena Dascalu, Cristina Iordache, Irina Gradinaru, Arina Alice Ciocan Pendefunda, Cezar Ilie Foia, Roxana Ionela Vasluianu

**Affiliations:** 1 Department of Implantology, Removable Dentures, Technology, Faculty of Dental Medicine, “Grigore T. Popa” University of Medicine and Pharmacy, 16 Universității Street, 700115 Iasi, Romania; magda.antohe@umfiasi.ro (M.-E.A.); monica.tatarciuc@umfiasi.ro (M.S.T.); marina.iordache@umfiasi.ro (C.I.); irina.gradinaru@umfiasi.ro (I.G.); roxana.vasluianu@umfiasi.ro (R.I.V.); 2 Department of Medical Informatics and Biostatistics, Faculty of Medicine, “Grigore T. Popa” University of Medicine and Pharmacy, 16 Universității Street, 700115 Iasi, Romania; 3 Department of Odontology-Periodontology, Fixed Prosthodontics, Faculty of Dental Medicine, “Grigore T. Popa” University of Medicine and Pharmacy, 16 Universității Street, 700115 Iasi, Romania; alice.ciocan@umfiasi.ro; 4 Department of Surgery II—Orthopedics and Traumatology, Faculty of Medicine, “Grigore T. Popa” University of Medicine and Pharmacy, 16 Universității Street, 700115 Iasi, Romania; foia.cezar-ilie@d.umfiasi.ro

**Keywords:** soft skills, dentistry, dentist–patient communication, digitalization

## Abstract

The quality of dental care is determined not only by the dentist’s technical competence but also by their ability to communicate effectively, listen actively, and establish a relationship of trust with the patient. Patients’ perceptions of dentists’ soft skills represent an essential component of patient-centered care; however, evidence from Romania remains limited. **Aim**: To evaluate patients’ perceptions of the importance of dentists’ non-technical (soft) skills in private dental practices and to analyze the influence of socio-demographic characteristics on these perceptions. **Materials and Methods**: The study included 297 patients attending private dental practices in Iași, Romania, who anonymously completed a questionnaire consisting of 20 items rated on a five-point Likert scale. Statistical analysis was performed using SPSS Statistics version 31.0. The Chi-square test and Fisher’s exact test were applied where appropriate. **Results**: Communication and interpersonal skills were considered the most important competencies by patients (91.3%), followed by cognitive skills (84.2%) and personal characteristics (80.2%), whereas artistic skills received the lowest level of appreciation (49.9%). Communication and interpersonal skills were the most highly valued domain (91.3%), followed by cognitive skills (84.2%) and personal characteristics (80.2%), whereas artistic skills received the lowest ratings (49.9%). Younger patients, urban residents, individuals with higher educational attainment, and those attending regular dental check-ups tended to assign greater importance to several of the non-technical competency domains assessed. The most pronounced age-related difference concerned digital technologies, with acceptance of telemedicine and digital communication decreasing markedly with increasing age. **Conclusions**: Communication and interpersonal skills were the non-technical competencies most highly valued by patients. The observed differences according to socio-demographic characteristics support the need for an individualized communication approach in dental practice.

## 1. Introduction

The quality of healthcare is determined not only by the accuracy of diagnosis and the effectiveness of therapeutic interventions but also by the quality of the relationship established between the clinician and the patient, a fundamental determinant of therapeutic outcomes [1,2]. In the present study, “non-technical competencies” is used as the overarching term for the interpersonal, cognitive, personal, ethical, managerial, and related non-clinical dimensions assessed from the patients’ perspective. At the core of this relationship lies effective communication, which facilitates mutual understanding, fosters trust, and creates the conditions for genuine collaboration throughout the therapeutic process. Beyond the mere exchange of medical information, communication enables patients to feel heard, understood, and actively involved in decisions regarding their own health, thereby contributing to the overall quality of care [3,4].

In dentistry, the role of communication is particularly important because dental consultations and treatments are frequently accompanied by fear, anxiety, and anticipation of pain. For many patients, dental care is associated with a perceived loss of control and an increased sense of vulnerability, factors that may influence both the decision to seek care and attitudes toward the recommended treatment [5,6]. Under these circumstances, the way dentists explain procedures, respond to patients’ questions, and address their concerns becomes an essential component of care, directly influencing patients’ psychological comfort and their willingness to cooperate throughout treatment [7,8].

Numerous studies have shown that effective dentist–patient communication is associated with reduced anxiety, improved treatment adherence, and higher levels of patient satisfaction. Consequently, communication is now widely recognized as one of the fundamental components of contemporary dental practice, contributing not only to the optimization of the therapeutic relationship but also to improved clinical outcomes [9,10].

A recent review of the international literature, published in 2025, further supports and expands this evidence by demonstrating that effective communication not only reduces anxiety and strengthens patient trust but also improves diagnostic accuracy and enhances patients’ understanding of their oral health status and the therapeutic options available to them [11].

In addition to clinical expertise, the practice of dentistry requires the development of a range of essential soft skills that support an effective therapeutic relationship. These include empathic communication, active listening, patience, respect for patients’ dignity and privacy, and the ability to adapt medical information to each patient’s level of understanding, thereby enabling informed participation in treatment-related decision-making [1,12]. Although communication skills were long regarded as complementary to clinical training, they are now widely recognized as an indispensable component of professional dental practice. Evidence consistently demonstrates that inadequate communication is associated with higher levels of patient anxiety, reduced trust in the dentist, and poorer adherence to recommended treatment [13]. The direct therapeutic value of soft skills extends far beyond the notion of good professional practice, positioning them as core clinical competencies that should be explicitly incorporated into undergraduate and postgraduate dental education curricula [14,15,16].

Patients’ perspectives on these competencies are equally—if not more—important than those of healthcare professionals, as patients are ultimately the ones who evaluate the quality of the clinical encounter and decide whether to return to the same dental practice or recommend the dentist to others. Studies conducted among older adults have shown that, when choosing a dentist, professional behavior and psychosocial skills often carry greater weight than the technical equipment available in the dental office. Moreover, the importance attributed to these competencies varies systematically according to patients’ age, sex, and place of residence [17,18]. However, these demographic differences in the perception of soft skills remain insufficiently documented among the adult population in Romania, where most available research has focused on the perspectives of dental students or practitioners rather than those of patients themselves [19,20].

Closely related to the effectiveness of dentist–patient communication is oral health literacy, defined as an individual’s ability to access, understand, evaluate, and use oral health information to make informed decisions regarding disease prevention and treatment. Recent research highlights the multidimensional nature of this concept and its important role in influencing clinical outcomes and reducing inequalities in oral healthcare [21,22].

Patients with limited oral health literacy often experience difficulties understanding medical information, expressing their concerns and expectations, and actively participating in the decision-making process [22,23]. Under these circumstances, the dentist’s ability to tailor information to each patient’s level of understanding becomes a fundamental requirement of clinical practice. Providing explanations in clear and accessible language, confirming patients’ understanding, and encouraging open dialogue are not merely indicators of courteous communication but essential elements of patient-centered care that ensure equitable access to high-quality oral healthcare, regardless of an individual’s level of oral health literacy [24,25].

Another aspect that has become increasingly relevant in contemporary dentistry and in discussions surrounding the quality of the dentist–patient relationship is the digitalization of dental practice, ranging from online appointment scheduling to telemedicine applications used for remote consultations, history taking, and patient counseling. However, acceptance of these technologies varies considerably across age groups. The literature indicates that the adoption of digital health technologies is influenced by perceived usefulness, digital self-efficacy, and privacy concerns, factors that affect younger and older adults differently [5]. Furthermore, patients’ place of residence and educational attainment are recognized as important determinants of satisfaction with dental care. Studies conducted in North America have identified significant disparities between urban and rural populations with respect to both access to dental services and expectations regarding the quality of interactions with dental professionals [6]. However, demographic differences in patients’ perceptions of dentists’ non-technical competencies remain insufficiently documented in the Romanian adult population, particularly in the context of private dental practice. This represents the principal knowledge gap addressed by the present study.

Against this background, the present study aimed to examine, from a patient-centered perspective, how patients attending private dental practices in Iași perceive the importance of different categories of dentists’ non-technical competencies and to assess whether these perceptions differ according to patients’ demographic characteristics, including sex, age, place of residence, educational level, and the frequency of routine dental check-ups. While the finding that communication and interpersonal skills are highly valued by patients is consistent with the international literature [3,11], the present study provides systematic, demographically stratified evidence on patients’ perceptions of dentists’ non-technical competencies in the context of Romanian private dental practices. The findings may contribute to strengthening patient-centered dental practice by providing dentists with empirically grounded evidence to support the adaptation of communication strategies to the individual characteristics and needs of each patient.

Based on the existing literature, we hypothesized that communication and interpersonal skills would be rated as the most important non-technical competency domain and that younger patients, urban residents, individuals with higher educational attainment, and those attending regular dental check-ups would assign greater importance to most of the non-technical competency domains assessed compared with their respective counterparts.

## 2. Materials and Methods

Participants: We recorded the opinions of 297 patients attending private dental practices in Iași, Romania, regarding the non-technical competencies considered important in dentist–patient interactions. The study was conducted between 2024 and 2026 in 18 private dental practices located in different areas of Iași, Romania. The inclusion of dental practices from different urban areas was intended to ensure a socio-demographically heterogeneous patient population and to avoid concentrating recruitment within a single area of the city. Adult patients attending the participating dental practices for dental consultations or treatment were eligible for inclusion if they were able to understand the purpose of the study and the content of the questionnaire, complete the questionnaire independently, and demonstrate an adequate level of cooperation. Participation was voluntary and required informed consent. Patients who declined to participate or were unable to adequately understand and complete the questionnaire were excluded.

Taking in consideration the resident population of Iași County (760,774 inhabitants, 2021 Romanian census), based on Cochran’s formula for proportion estimation with 95% confidence and maximum variance (*p* = 0.5), a sample of 384 subjects would be required to achieve a ±5% margin of error at the county-population level. That is why we initially established as target for our sample 400 participants. Of the 400 patients initially invited to participate, 76 declined (response rate = 81.0%) and 324 agreed to participate to the study; of these, 27 questionnaires were excluded due to incomplete data, resulting the final sample of patients (74.25% of those initially invited). We obtained therefore 297 participants in our study, which correspond to a margin of error of approximately ±5.7%; this is reasonably close to, though slightly above the conventional threshold. Anyway, the true target population of our study was not the general county population, but only adult patients attending private dental practices in Iasi, for which no official count exists; it follows that the sample size we reached was large enough to achieve the usual standard of ±5% margin of error.

The patients were recruited based on voluntary participation; the study sample was therefore selected through convenience sampling.

Data collection: Patients were asked to fill anonymously a 20-items questionnaire in which they expressed their extent of agreement with different statements regarding non-technical competencies and related aspects of dental care during dentist–patient interactions (Appendix A); the agreement extent was expressed using a Likert scale from 1 to 5, where 1 corresponded to “very small extent” and 5 to “very large extent”; all items were weighted equally and there was no case of reverse-coded items. The questionnaire aimed to evaluate 6 specific soft skills, as follows: cognitive skills (items 4, 13, 17); communication and interpersonal skills (items 3, 7, 8, 11, 12, 18, 19), artistic skills (item 9), ethical and professional skills (items 2, 6, 14, 16, 20), personal features (items 1, 10) and management skills (items 5, 15). These 6 soft skills were expressed as numerical scores (calculated as the sums of the items involved) which were further transformed in ordinal variables according to the scales indicated in Table A1 (Appendix A). The questionnaire was presented and explained separately to each subject, along with the research goals.

Questionnaire: The questionnaire was adapted and developed starting from the models already existent in the scientific literature. The questionnaire used in the present study was developed within the Erasmus+ project “Exploring, Assessing and Applying SOFT Skills in DENTAL Medicine University Students” (DENTA_SOFT), No. 2022-1-BG01-KA220-HED-000085701. Within the framework of the project, distinct instruments were developed to assess non-technical (soft) skills from different perspectives. Thus, in addition to the instrument designed for dental students, a separate questionnaire was specifically and explicitly developed for patients, focusing on their perceptions and expectations regarding dentists’ non-technical skills in the context of dentist–patient interactions. The patient-specific instrument was developed by a multidisciplinary team of specialists, including dental professionals and psychologists, to ensure the relevance, clarity, and appropriateness of the items to the patients’ perspective and experience. Its clarity and relevance were assessed by a team of 25 international specialists, by calculating the Inter-rater Agreement (IRA), Item Content Validity (ICV) and Scale-Content Validity (SCV) indexes, and its reliability was assessed by calculating the alpha-Cronbach index. The minimal value of IRA was 72.0% for clarity (in case of items 11, 13 and 17 of the questionnaire) and also 72.0% for relevance (in case of items 5, 7 and 17 of the questionnaire); the minimal value of ICV was 88.0% for clarity (in case of items 2, 6, 10, 12, 13, 14 and 17 of the questionnaire) and 84.0% for relevance (in case of item 5 of the questionnaire); the SCV was 91.6% for clarity and 89.4% for relevance. In order to calculate the questionnaire’s reliability, it was tested at first on a pilot sample made from 20 patients; the global alpha-Cronbach index was 0.892; the alpha-Cronbach indexes for the 6 sections of the questionnaire, corresponding to the 6 categories of soft skills, were the following: 0.834 for cognitive skills section; 0.785 for communication and interpersonal skills section; 0.769 for ethical and professional skills section; 0.726 for personal features section and 0.837 for management skills; the artistic skills were evaluated through a single item, so alpha-Cronbach coefficient was not applicable. On these bases, the questionnaire was considered suitable for being applied in our study.

The questionnaire was based on an integrative and multidimensional conceptual approach to the non-technical aspects of dental care. In addition to dentists’ non-technical competencies, selected items address individual, environmental, and organizational factors that may influence patients’ perceptions and experiences of dental care. These factors were considered in relation to, rather than as direct components of, dentists’ soft skills, reflecting the broader context in which dentist–patient interactions take place.

Variables: The questionnaire’s items were grouped in order to calculate 6 different scores, each one of them corresponding to a specific category of soft skills: cognitive skills, communication and interpersonal skills, artistic skills, ethical and professional skills, personal features and management skills. Other variables included in the study were the basic demographic features (sex, age, residence, education level), as well as the frequency of dental controls.

Statistical analysis: The data from the questionnaire were recorded in a data file in SPSS 31.0 (SPSS Inc., Chicago, IL, USA) for Windows. The answers to each item were characterized through frequency distributions and contingency tables. The numerical variables were characterized through descriptive statistics (mean, standard deviation, range, median and IQR). The comparisons between samples were performed using the Chi-squared test (with Fisher correction when necessary). Effect sizes were also calculated, using Cramer’s V coefficient, in order to assess the practical significance of the associations between variables, independently of statistical significance. Effect sizes were interpreted according to the conventional scale: V < 0.10 small; 0.10 ≤ V< 0.30 medium; V ≥ 0.30 large. Given the large number of comparisons performed across the demographic features (20 items of the questionnaire and 6 aggregated soft skills scores) it was necessary to apply the Bonferroni correction for multiple testing, in order to reduce the risk of Type I error. The correction was applied separately for each family of tests: α = 0.05/20 = 0.0025 for comparisons involving the 20 individual items of the questionnaire, and α = 0.05/6 = 0.0083 for comparisons involving the 6 aggregate scores; this approach was chosen because the analysis is performed on two conceptually distinct levels—item-level versus aggregated score-level differences, and applying a single, undifferentiated correction across all comparisons would not adequately reflect this distinction. Results were reported as statistically significant only when they met the corrected threshold for their corresponding family. For narrative purposes only, in the Results section in some cases the response categories 4 (“to a large extent”) and 5 (“to a very large extent”) were combined into a single “agreement” category, in order to facilitate the interpretation and to highlight better the differences between the compared subgroups; for this unified category the percentages were reported with their 95% confidence intervals (Wilson method) as well. The full five-categories distribution of responses is, however, reported in full for the overall sample, both at item level and for the aggregate scores, as well as for all demographic subgroup comparisons. 

Ethical statement: Participation in the study was voluntary. The subjects were informed about the study and the content of the questionnaire, and they agreed with the informed consent. The questionnaires were filled anonymously in order to protect the subjects’ intimacy and to obtain objective answers as much as possible. The study was approved by the Ethical Committee of “Grigore T. Popa” University of Medicine and Pharmacy from Iasi, Romania (decision no. 427/07.04.2024).

## 3. Results

The study population consists mainly of women (59.3%) living in urban areas (69.4%), with three-quarters of the patients under the age of 55 (73.0%); 40.7% of the patients have a college degree, and 29.6% have a high school diploma. One-quarter of the patients (26.6%) visit the dentist only in emergencies, and another 20.5% visit the dentist less than once a year; the rest undergo dental checkups once or twice a year—Table 1.

A detailed analysis of patients’ responses to the questionnaire shows that the attributes they most frequently desire from their dentist during their interaction with him or her (in over 80% of cases) are as follows:

- Respect for the patient and his or her privacy (item 2)—88.2%;
- A calm and confident demeanor (item 1)—87.6%;
- Attention to detail and performing dental procedures calmly and without rushing (item 13)—86.8%;
- A friendly and calm attitude from the entire dental office team (item 12)—84.6%;
- Providing detailed explanations about the patient’s dental condition and treatment options (item 4)—84.1%;
- Patience and willingness to listen to the patient’s health concerns (item 3)—83.1%.

At the other extreme, just under one-third of patients (31.0%) prefer to visit larger dental clinics rather than individual dental practices (item 20). Similarly, just over one-third of patients prefer that the dentist not wear a mask outside of the actual treatment period (item 19)—34.0% state that they are not afraid when the dentist uses a laser instead of anesthesia (item 16)—38.4%—and that they can sometimes tolerate pain depending on the treatment (item 6)—38.7%—Table 2.

A summary of patients’ opinions regarding the non-technical competencies assessed in the present study is provided in Table 3 and Table 4, highlighting the following findings (Table 3 and Table 4):

- The skills considered most important by nearly all patients are communication and interpersonal skills (identified as such by 91.3% of patients and rated with a mean score of 27.26 ± 4.656 and a median of 28.00, ranging from 9 to 35);
- Next in line are cognitive skills, cited by 84.2% of patients (with a mean score of 11.86 ± 2.482 and a median of 12.00, ranging from 3 to 15), and personal characteristics, reported by 80.2% of patients (with a mean score of 8.05 ± 1.726 and a median of 8.00, ranging from 2 to 10);
- Management skills are important to 71.4% of patients (with a mean score of 7.50 ± 1.880 and a median of 8.00, ranging from 2 to 10), and professional ethics are important to 69.7% of them (with a mean score of 17.37 ± 3.598 and a median of 17.00, ranging from 5 to 25);
- Artistic skills are the least valued by patients, being considered important by only 49.9% of them (with a mean score of 3.40 ± 1.278 and a median of 3.00 within a range of 1 to 5).

**Table 3:** Summary of patients’ opinions on the non-technical competencies that dentists should apply in practice—qualitative assessment.

	The Importance of Soft Skills in Work Performance									
	Very Low		Low		Moderate		High		Very High	
	n	%	n	%	n	%	n	%	n	%
Cognitive Skills	3	1.0	6	2.0	38	12.8	119	40.1	131	44.1
Communication and Interpersonal Skills			6	2.0	20	6.7	152	51.2	119	40.1
Artistic Skills	31	10.4	40	13.5	78	26.3	75	25.3	73	24.6
Ethical and Professional Values	1	0.3	6	2.0	83	27.9	141	47.5	66	22.2
Personal Characteristics	2	0.7	7	2.4	50	16.8	100	33.7	138	46.5
Management Skills	5	1.7	15	5.1	65	21.9	116	39.1	96	32.3

**Table 4:** Summary of patients’ opinions on the non-technical competencies that dentists should apply in practice—quantitative assessment.

The Importance of Soft Skills in Work Performance	N	M ± SD	Min	Max	Median	IQR
Cognitive Skills	297	11.86 ± 2.482	3	15	12.00	10.00 ÷ 14.00
Communication and Interpersonal Skills	297	27.26 ± 4.656	9	35	28.00	24.00 ÷ 31.00
Artistic Skills	297	3.40 ± 1.278	1	5	3.00	3.00 ÷ 4.00
Ethical and Professional Values	297	17.37 ± 3.598	5	25	17.00	15.00 ÷ 20.00
Personal Characteristics	297	8.05 ± 1.726	2	10	8.00	7.00 ÷ 9.50
Management Skills	297	7.50 ± 1.880	2	10	8.00	6.00 ÷ 9.00

No statistically significant differences are found between sexes regarding specific responses to the questions in the questionnaire, even if some relevant discrepancies are identified (Appendix B, Table A2). For example, the proportion of women who prefer to receive from their dentist detailed explanations about the state of their teeth and treatment options (item 4, 86.4%) is obviously higher than the corresponding proportion of men (81.0%). Also, the proportion of women who value the advice given by the dentist regarding behaviors related to oral health (item 14, 81.3%) is much higher than the corresponding proportion of men (65.3%). Relevant sexes differences are also recorded for item 8: 74.5% of women highly appreciate when the dentist encourages them in their efforts to improve their oral hygiene, compared to only 63.6% of men.

For all other statements in the questionnaire, women express their agreement to a large or very large extent at higher percentages than men. Some relevant examples include the following: women more frequently than men appreciate the clear and simple language used by dentists, both spoken and written (item 7, 77.8% versus 68.6%), dental offices decorated in pastel or warm colors (item 9, 55.7% versus 41.3%), background music played during dental procedures (item 10, 63.1% vs. 53.7%), or when the dentist takes his or her time during procedures and pays attention to detail (item 13, 90.9% vs. 81.0%).

**Figure 1:**
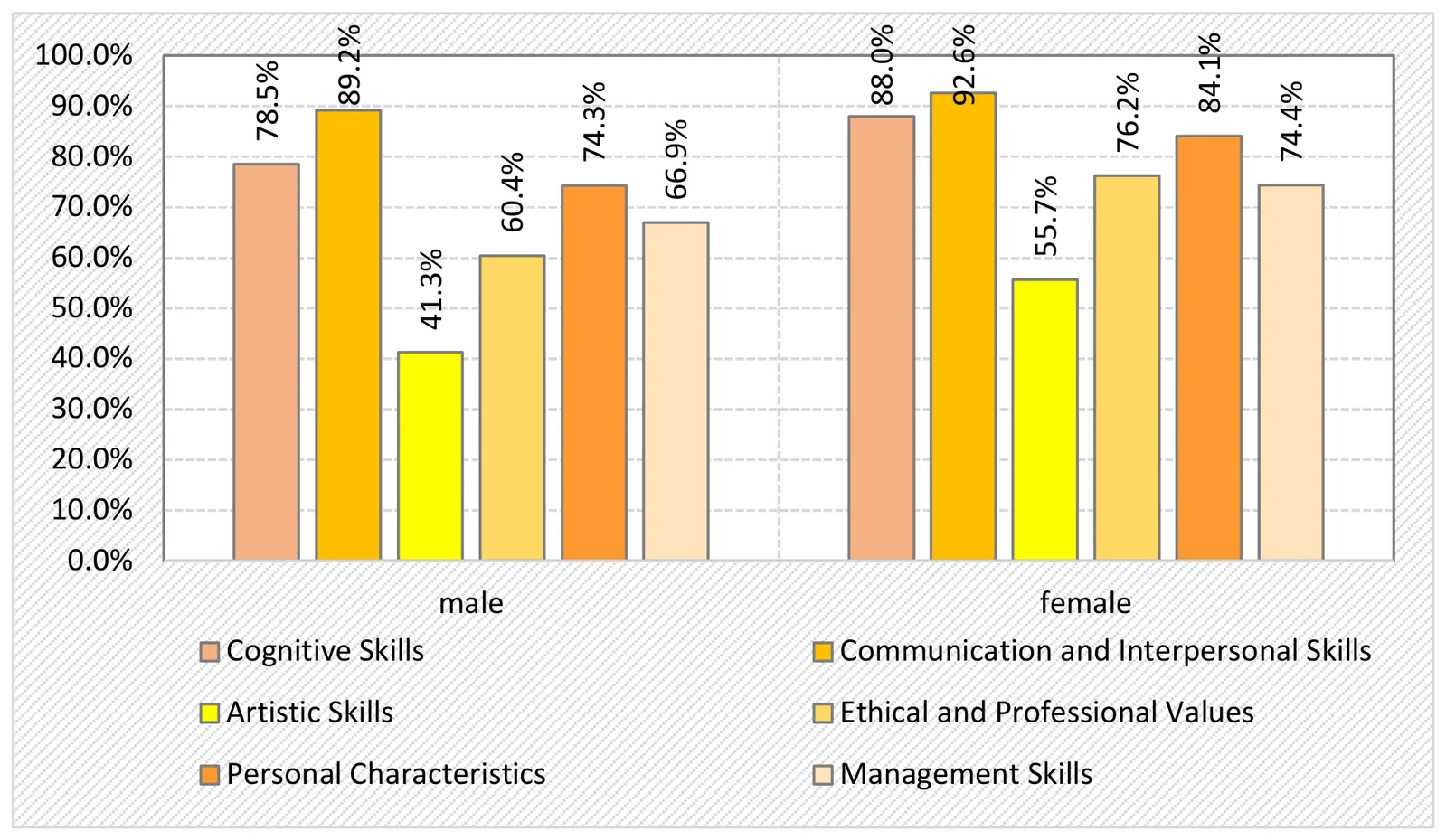
Percentage of patients rating each soft-skill domain as important (“high” or “very high”), by sexes.

Women rate all six types of surveyed soft skills more highly than men, even if the statistically significant differences are not recorded (Figure 1, Appendix B, Table A3). The ethical and professional values are rated as important or very important by 76.2% of women compared to only 60.4% of men. Similarly, the cognitive skills are rated as important or very important by 88.0% of women compared to 78.5% of men, as well as the personal characteristics, which are valued as high or very high by 84.1% of women versus 74.3% of men. Artistic abilities are also valued as high or very high more frequently by women than by men (55.7% versus 41.3%), and the smallest discrepancy is observed in communication and interpersonal skills, which are valued as high or very high by the vast majority of patients, regardless of their sex (92.6% of women and 89.2% of men).

With regard to ranking soft skills competencies in order of importance, male patients maintained the same order as that reported for the overall group (Figure 1), namely: communication and interpersonal skills (89.2%), followed by cognitive skills (78.5%), personal characteristics (74.3%), management skills (66.9%), ethical and professional values (60.4%), and artistic skills (41.3%). For women, however, the hierarchy shifts slightly: communication and interpersonal skills are still considered the most important (92.6%), followed by cognitive skills (88.0%) and personal characteristics (84.1%), followed by ethical and professional values (76.2%), which are ranked ahead of management skills (74.4%); artistic skills are again ranked last (55.7%).

The comparative study by age groups reveals statistically significant differences in patients’ opinions regarding the following items of the questionnaire (Appendix B, Table A4):

- Item 1: Over 90% of young patients, up to age 44, want their dentist to be a calm and confident person; this percentage drops to 85.7% among those aged 45–54, 76.2% among those aged 55–64, and rises to 81.5% among patients over 65 (*p* < 0.001 *, V = 0.202—average effect size);
- Item 2: Over 90% of patients aged 54 and under want their dentist to respect them and their privacy, compared to only 76.2% of those aged 55–64 and 81.6% of those over 65 (*p* = 0.002 *, V = 0.179—average effect size);
- Item 10: Over 60% of patients aged 54 and under feel more comfortable when background music is playing during dental procedures, a percentage that drops quite dramatically to about 45% among patients over 55 (*p* < 0.001 *, V = 0.195—average effect size);
- Item 17: 61.4% of younger patients, up to age 34, believe it is much more efficient for the dentist to contact them via digital communication platforms to schedule appointments and monitor them over the long term; this percentage also drops sharply as patients get older, to 46.5% of those aged 35–44, 36.5% of those aged 45–54, only 23.8% of patients aged 55–64, and only 15.8% of patients over 65 (*p* < 0.001 *, V = 0.230—average effect size);

Statistically significant differences are found between age groups regarding patients’ level of agreement with the following categories of investigated non-technical (soft skills) competencies (Appendix B, Table A5):

- Cognitive skills: are considered very important by approximately 90% of patients aged 54 and under, a percentage that drops to 66.6% among those aged 55–64 and 71.0% among those over 65 (*p* < 0.001 *, V = 0.243—average effect size);
- Communication and interpersonal skills: are also considered the most important by 94.8% of patients aged 34 and under, 98.3% of those aged 35–44, and 88.9% of those aged 45–54, a percentage that drops to about 84% among those over 55 (*p* = 0.002 *, V = 0.186—average effect size);
- Ethical and professional values: are considered most important among the 35–44 age group, where 82.8% of patients consider them important to a large or very large extent, compared with about 75% of patients aged 34 and under or between 45 and 54, and about 50% of those over 55 (*p* < 0.001 *, V = 0.188—average effect size);
- Personal characteristics: are considered very important among younger patients (87.5% of those aged 34 and under and 94.9% of those aged 35–44); this percentage drops to 70% among patients over 45 (*p* < 0.001 *, V = 0.214—average effect size).

Management skills are also considered important to a large or very large extent by about 80% of patients up to age 44, a percentage that drops to 68.3% among those aged 45–54, 54.7% among those aged 55–64, and rises to 60.5% among patients over 65. Artistic skills are generally not considered very important; the highest percentage of patients who consider them important or very important was found in the 35–44 age group (67.2%); only 48.0% of patients aged 34 and under consider these types of skills important, as do 49.2% of those aged 45–54, a percentage that drops to 42.9% among those aged 55–64 and 36.9% among those over 65.

**Figure 2:**
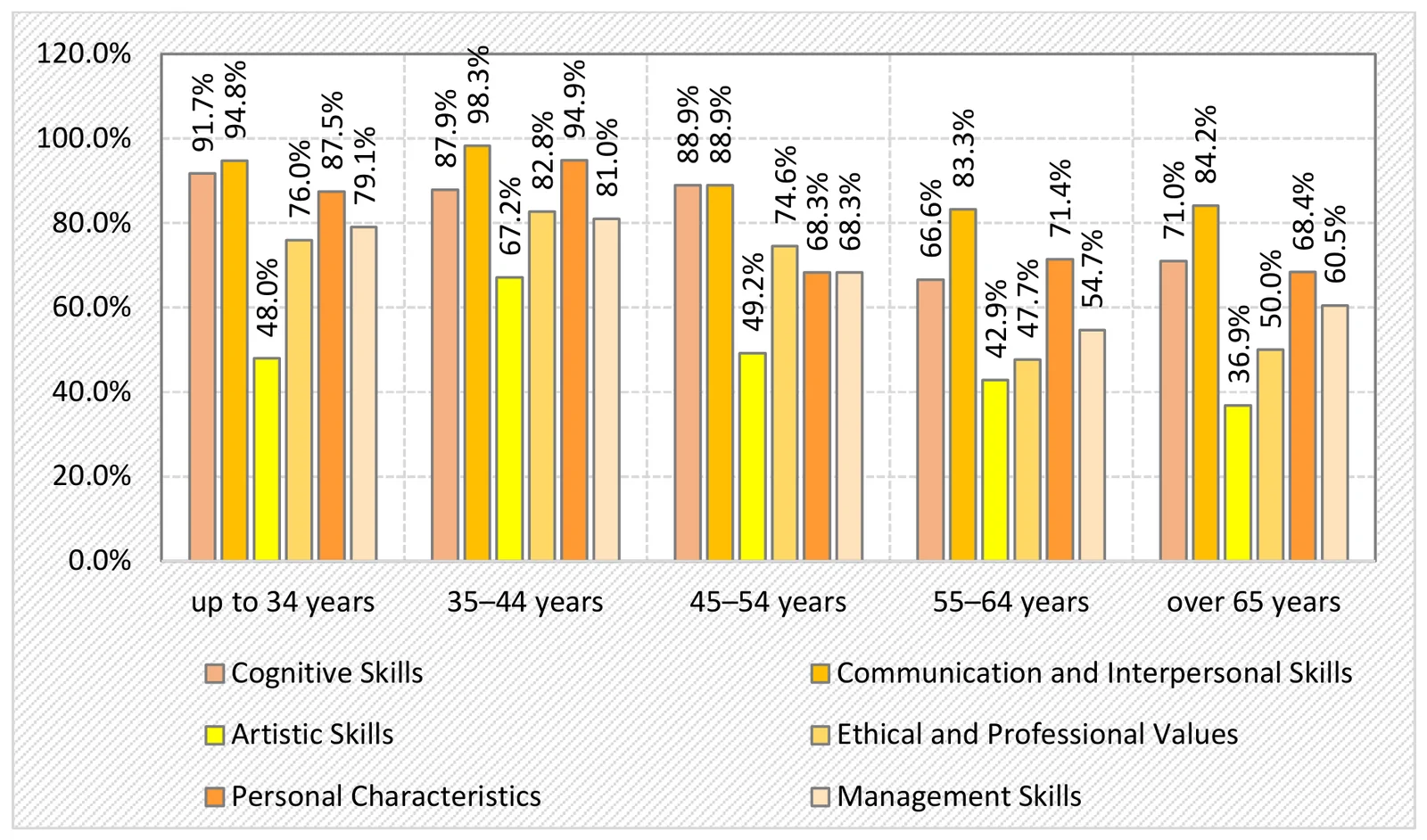
Percentage of patients rating each soft-skill domain as important (“high” or “very high”), by age groups.

In the age group up to 34 years old (Figure 2), the ranking of soft skills in order of importance for job performance is identical to that reported in the overall sample, namely, communication and interpersonal skills are the most important (94.8%), followed by cognitive skills (91.7%), personal characteristics (87.5%), management skills (79.1%), ethical and professional values (76.0%), and artistic abilities (48.0%). The same order was observed among patients over 65 years of age, who also ranked communication and interpersonal skills first (84.2%), followed by cognitive abilities (71.0%), personal characteristics (68.4%), management skills (60.5%), ethical and professional values (50.0%), and artistic abilities (36.9%).

In the 35–44 age group, communication and interpersonal skills are also ranked first (98.3%), but are followed by personal characteristics (94.9%), cognitive abilities (87.9%), ethical and professional values (82.8%), management skills (81.0%), and artistic abilities (67.2%).

In the 45–54 age group, communication and interpersonal skills also rank first (88.9%), tied with cognitive abilities (88.9%) and followed by ethical and professional values (74.6%), personal characteristics (68.3%), rated on par with management skills (68.3%) and followed by artistic skills (49.2%).

In the 55–64 age group, communication and interpersonal skills also rank first (83.3%), followed by personal characteristics (71.4%), cognitive abilities (66.6%), management skills (54.7%), ethical and professional values (47.7%), and artistic abilities in last place (42.9%).

Patients’ level of agreement with the statements in the questionnaire is consistently higher in urban areas than in rural areas for some items in the questionnaire (Appendix B, Table A6); the differences observed are statistically significant in the following cases:

- Item 2: 92.2% of urban patients want their dentist to respect them and their privacy, compared to 79.2% of rural patients (*p* < 0.001 *, V = 0.255—average effect size);
- Item 4: 91.7% of urban patients believe that the dentist should provide them with detailed explanations about their dental condition and treatment options, compared to only 67.1% of rural patients (*p* < 0.001 *, V = 0.339—large effect size);
- Item 5: 84.0% of urban patients consider it very important for the dentist to be punctual, compared to only 69.3% of rural patients (*p* < 0.001 *, V = 0.273—average effect size);
- Item 13: Urban patients are more likely than rural patients to want the dentist to take their time during procedures and pay attention to detail (91.8% vs. 75.8%) (*p* = 0.001 *, V = 0.248—average effect size);
- Item 14: Urban patients appreciate it more often than rural patients when the dentist provides additional advice on oral health behaviors (82.1% vs. 58.3%) (*p* < 0.001 *, V = 0.272—average effect size);
- Item 15: Urban patients are more likely than rural patients to want their dentist to be familiar with telemedicine systems (57.8% vs. 34.1%) (*p* = 0.002 *, V = 0.243—average effect size).

Statistically significant differences were found between the patients’ places of residence regarding their level of agreement with the following categories of non-technical (soft skills) competencies investigated (Appendix B, Table A7):

- Cognitive skills, which are considered important or very important by 92.8% of patients in urban areas, compared to 64.9% of those in rural areas (*p* < 0.001 *, V = 0.393—large effect size);
- Communication and interpersonal skills, which are also considered the most important by 94.7% of patients in urban areas, compared to 83.5% of those in rural areas (*p* < 0.001 *, V = 0.252—average effect size);
- Personal characteristics, which are considered important or very important by 86.9% of urban patients, compared to 64.9% of rural patients (*p* < 0.001 *, V = 0.284—average effect size);
- Management skills, which are also considered important or very important by 79.1% of urban patients, compared to 53.9% of rural patients (*p* < 0.001 *, V = 0.330—large effect size).

Artistic abilities are considered the most important by 53.4% of urban patients, compared to 41.8% of rural patients, while ethical and professional values are considered important or very important by 75.8% of urban patients, compared to 56.1% of rural patients.

**Figure 3:**
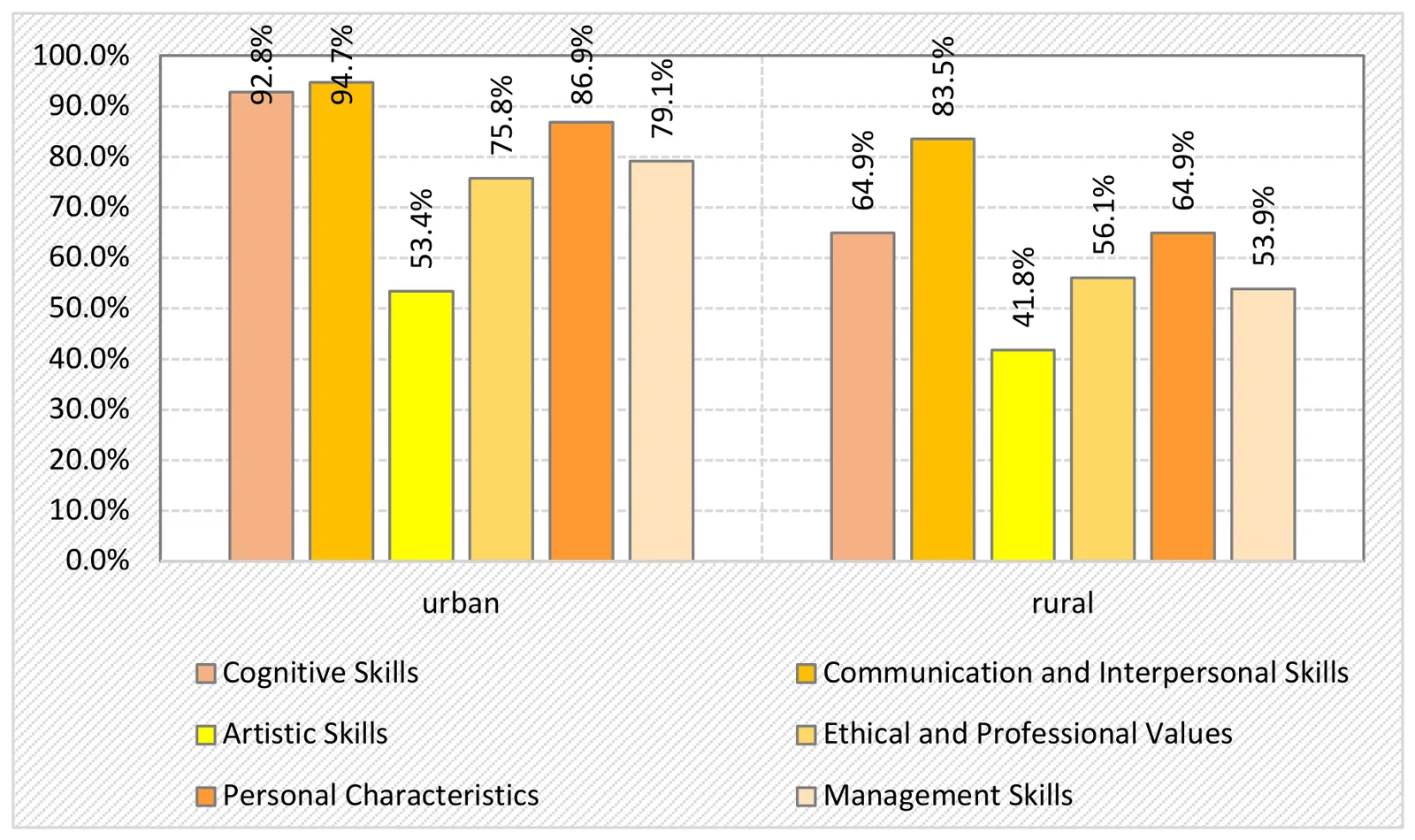
Percentage of patients rating each soft-skill domain as important (“high” or “very high”), by place of residence.

With regard to the ranking of soft skills competencies in order of importance (Figure 3), in urban areas this ranking is identical to that observed in the overall sample: communication and interpersonal skills are considered the most important (94.7%) and are followed by cognitive skills (92.8%), personal characteristics (86.9%), management skills (79.1%), ethical and professional values (75.8%), and artistic abilities (53.4%).

In rural areas, however, the order of competencies shifts slightly: communication and interpersonal skills remain in first place (83.5%), followed by cognitive skills (64.9%), which are rated on par with personal characteristics (64.9%); in the next position, however, are ethical and professional values (56.1%), followed by management skills (53.9%) and artistic abilities (41.8%).

In general, patients who are college or university graduates show a higher degree of agreement with the statements in the questionnaire compared to patients who are high school graduates or have less education (Appendix B, Table A8). In case of a singular item (item 1), the differences observed are statistically significant: 91.8% of university graduates and 90.0% of college graduates want their dentist to be a calm and confident person, compared to 86.4% of high school graduates and only 73.7% of those with no formal education (*p* < 0.001 *, V = 0.210—average effect size).

Among the soft skills competencies examined, statistically significant differences were found in patients’ opinions based on their level of education in two cases (Appendix B, Table A9): cognitive skills and communication and interpersonal skills.

Thus, 91.7% of patients with a university degree, as well as 86.0% of college graduates and 79.5% of high school graduates, believe that dentists’ cognitive skills are very important to their job performance, compared to only 68.4% of patients with no formal education (*p* = 0.003 *, V = 0.182—average effect size). Furthermore, 92.6% of university graduates and 96.0% of college graduates, as well as 89.7% of high school graduates, share the same opinion regarding communication and interpersonal skills, compared to 84.3% of patients with no formal education (*p* = 0.006 *, V = 0.161—average effect size).

No statistically significant differences were found for the other categories of soft skills competencies assessed. Patients’ opinions regarding artistic abilities are reserved; 54.6% of university graduates, as well as 55.3% of patients with no formal education, consider them important to a dentist’s job performance, compared to only 46.0% of college graduates and only 43.2% of high school graduates. 85.1% of university graduates and 84.0% of college graduates, as well as 79.5% of high school graduates, rate the dentist’s personal characteristics as very important to their performance, compared to only 60.5% of patients with no formal education. Ethical and professional values are considered very important for job performance by 76.1% of patients who are university graduates, compared to nearly 70% of high school or college graduates and only 52.7% of patients with no formal education; similarly, management skills are considered very important by college graduates (78.0% of them), compared to only 67.0% of high school graduates and only 60.5% of patients with no formal education.

**Figure 4:**
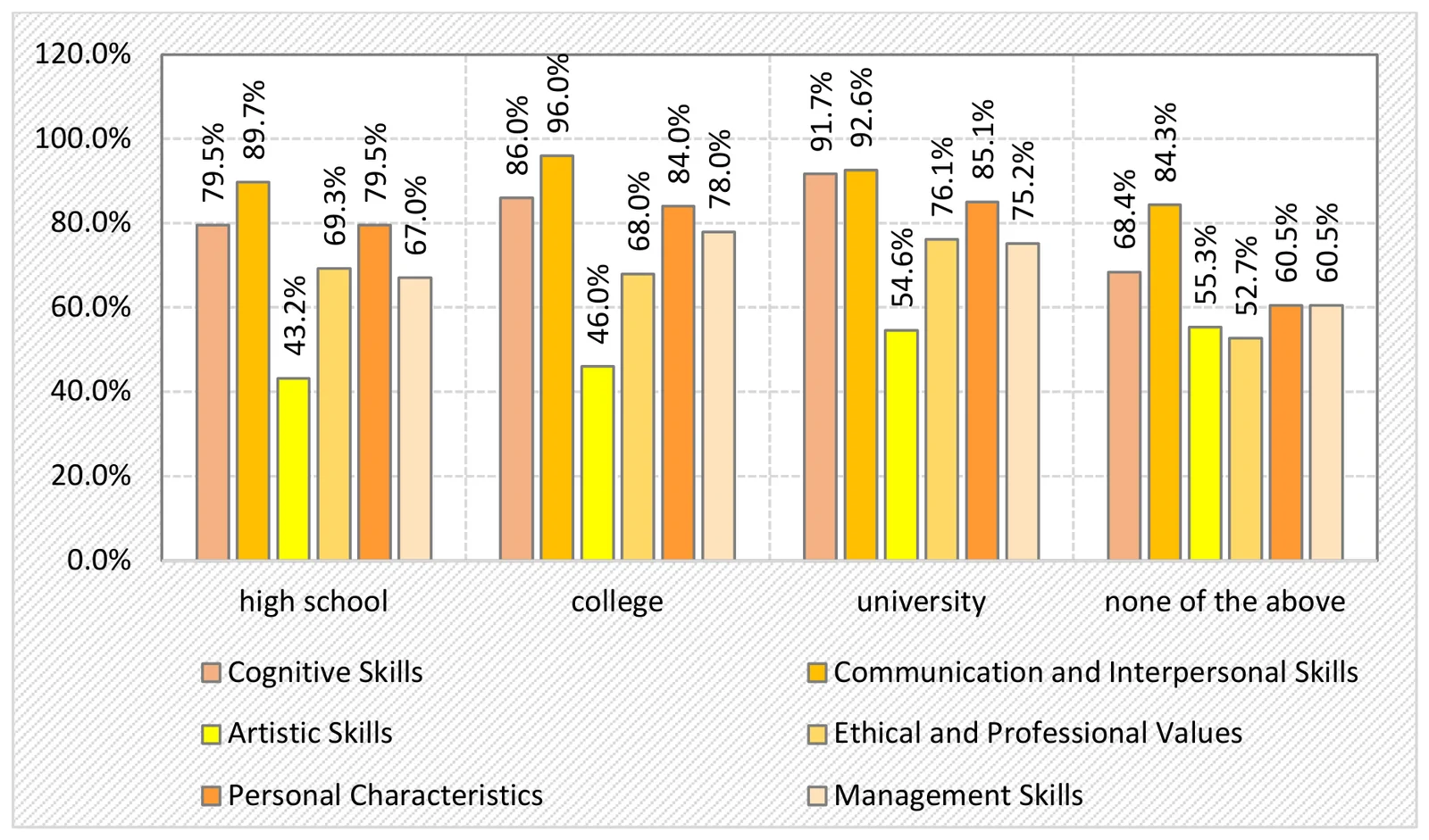
Percentage of patients rating each soft-skill domain as important (“high” or “very high”), by educational level.

University graduates (Figure 4) rank communication and interpersonal skills first in order of importance among soft skills (92.6%), followed by cognitive skills (91.7%), personal characteristics (85.1%), ethical and professional values (76.1%), management skills (75.2%), and artistic abilities (54.6%).

College graduates also rank communication and interpersonal skills first (96.0%), followed by cognitive skills (86.0%), personal characteristics (84.0%), management skills (78.0%), ethical and professional values (68.0%), and artistic abilities (46.0%)—this order is identical to that reported for the overall group.

High school graduates rank communication and interpersonal skills first (89.7%), followed by cognitive skills (79.5%) and personal characteristics, which are rated equally (79.5%), ethical and professional values (69.3%), management skills (67.0%), and artistic abilities (43.2%)—this ranking is, in fact, identical to that of college graduates.

Finally, patients without formal education also rank communication and interpersonal skills first (84.3%), followed by cognitive abilities (68.4%), personal characteristics (60.5%), management skills—also rated equally (60.5%)—and artistic abilities (55.3%), while ethical and professional values are ranked last in the hierarchy (52.7%).

The highest levels of agreement with the statements surveyed in the questionnaire were recorded among patients who visit the dentist every 6 months or once a year, while at the other extreme were patients who visit the dentist only in cases of emergency (Appendix B, Table A10). The differences between these patient categories are statistically significant in case of the following items:

- Item 1: 91.6% of patients who have dental checkups every 6 months and 95.9% of those who have such checkups once a year want their dentist to be a calm and confident person, compared to only 73.4% of those who visit the dentist only in emergencies (*p* < 0.001 *, V = 0.204—average effect size);
- Item 4: 94.9% of patients who have dental checkups every 6 months and 91.9% of those who have such checkups once a year want their dentist to provide detailed explanations about their dental condition and treatment options, compared to only 80.4% of those who go for checkups less than once a year and 69.6% of those who see a dentist only in emergencies (*p* = 0.001 *, V = 0.191—average effect size);
- Item 8: 88.1% of patients who have dental checkups every 6 months and 78.6% of those who have such checkups once a year appreciate it when their dentist encourages them in their efforts to improve their oral hygiene, compared to only 55.7% of those who go for checkups less than once a year and 57.0% of those who see a dentist only in emergencies (*p* < 0.001 *, V = 0.234—average effect size);
- Item 10: 76.3% of patients who have dental checkups every 6 months and 68.4% of those who have such checkups once a year feel more comfortable when background music is playing during dental procedures, compared to about 45% of those who see a dentist less than once a year or only in emergencies (*p* < 0.001 *, V = 0.199—average effect size);
- Item 17: 52.6% of patients who have dental checkups every 6 months and 49.0% of those who have such checkups once a year believe it is much more efficient for the dentist to contact them via digital communication platforms to schedule appointments, compared to only 27.9% of those who go for checkups less than once a year and 36.7% of those who see a dentist only in emergencies (*p* = 0.002 *, V = 0.188—average effect size).

In which concerns the 6 categories of soft skills competencies investigated, statistically significant differences were found in patients’ opinions based on the frequency of their visits to the dentist in the following cases (Appendix B, Table A11):

- Cognitive skills: 93.2% of patients who undergo dental checkups every 6 months, as do 93.9% of those who have such checkups once a year, consider cognitive skills to be very important in a dentist’s professional performance, compared to only 75.4% of those who go for checkups less than once a year and 72.1% of those who see a dentist only in emergency cases (*p* < 0.001 *, V = 0.214—average effect size);
- Communication and interpersonal skills: 94.9% of patients who have dental checkups every 6 months, as well as 96.0% of those who have such checkups once a year and 90.1% of those who go for checkups less than once a year consider communication and interpersonal skills to be very important to a dentist’s performance, compared to 83.5% of those who see a dentist only in emergencies (*p* < 0.001 *, V = 0.195—average effect size);
- Personal characteristics: 91.5% of patients who have dental checkups every 6 months, as do 91.9% of those who have such checkups once a year, consider personal characteristics to be very important in a dentist’s performance, compared to only 72.1% of those who go for checkups less than once a year and 63.3% of those who see a dentist only in emergencies (*p* < 0.001 *, V = 0.235—average effect size);
- Management skills: are considered very important to a dentist’s performance by 84.8% of patients who have dental checkups every 6 months, 75.5% of those who have such checkups once a year, and only 62.2% of those who go for checkups less than once a year and 63.3% of those who see a dentist only in emergencies (*p* = 0.005 *, V = 0.179—average effect size).

Ethical and professional values and management skills are considered slightly less important. They are considered very important to a dentist’s performance by 83.1% of patients who have dental checkups every 6 months, 74.5% of those who have such checkups once a year, compared to only 55.7% of those who go for checkups less than once a year and 64.6% of those who see a dentist only in emergencies. Artistic skills are considered the least important. Only 52.5% of patients who have dental checkups every 6 months and 58.2% of those who have such checkups once a year consider them very important to the dentist’s performance, percentages that drop to 45.9% among those who go for checkups less than once a year and 40.5% among those who see a dentist only in emergencies.

**Figure 5:**
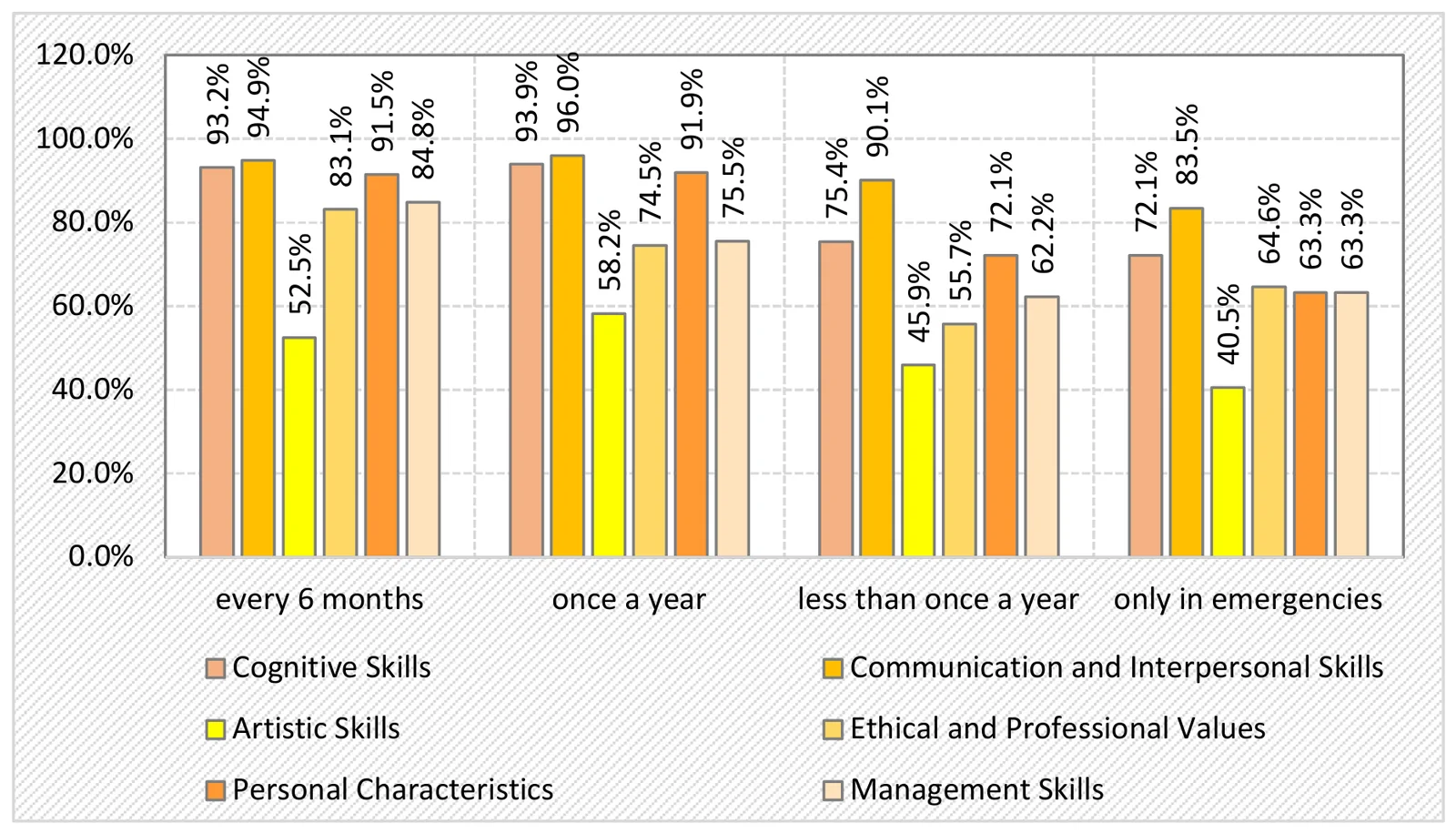
Percentage of patients rating each soft-skill domain as important (“high” or “very high”), by frequency of dentist appointments.

The ranking of soft skills competencies by importance (Figure 5) is identical to that observed in the overall cohort among patients who attend checkups every 6 months, once a year, and less than once a year.

Thus, for patients who visit the dentist every 6 months, the most important category of soft skills competencies is communication and interpersonal skills (94.9%), followed by cognitive skills (93.2%), personal characteristics (91.5%), management competencies (84.8%), ethical and professional values (83.1%), and artistic skills (52.5%).

For patients who visit the dentist for a checkup once a year, the most important category of soft skills competencies is also communication and interpersonal skills (96.0%), followed by cognitive skills (93.9%), personal characteristics (91.9%), management skills (75.5%), ethical and professional values (74.5%), and artistic skills (58.2%).

The same ranking holds true for patients who visit the dentist for a checkup less than once a year. In their case, communication and interpersonal skills are considered important by 90.1% of patients, followed by cognitive skills (75.4%), personal characteristics (72.1%), management skills (62.2%), ethical and professional values (55.7%), and artistic abilities (45.9%).

For patients who visit the dentist only in emergency situations, the ranking of soft skills competencies is slightly different. Communication and interpersonal skills still rank first in order of importance (83.5%), followed by cognitive skills (72.1%), ethical and professional values (64.6%), personal characteristics (63.3%)—rated on par with management skills (63.3%)—and artistic abilities (40.5%).

## 4. Discussion

### 4.1. Principal Findings

The present study identified several key findings regarding patients’ perceptions of non-technical competencies in dental care. These findings provide a clear and consistent picture of patients’ expectations regarding dentists’ non-technical (soft) skills. Overall, the statistically significant results identified in our study are consistently accompanied by at least moderate effect sizes—which means that their statistical significance is also practical and reflects a clinical relevance; conversely, no non-significant results are associated with large effect sizes, which means that the Bonferroni correction did not obscure any comparison with a substantial effect.

Communication and interpersonal skills emerged as the most highly valued domain, followed by cognitive skills and personal characteristics, whereas artistic skills received the lowest ratings. Differences in patients’ perceptions were also observed according to age, area of residence, educational attainment, and frequency of dental attendance. Overall, these findings indicate that patients assign particular importance to the relational and communicative dimensions of dental care, while also showing that perceptions of non-technical competencies vary across sociodemographic and dental attendance groups.

### 4.2. Comparison with Previous Literature

Our results are consistent with trends reported in the international literature while also offering insights specific to the Romanian context.

The most important finding of this study is that patients ranked communication and interpersonal skills as the most important competencies (91.3%), even ahead of cognitive skills (84.2%). This finding is consistent with the existing literature, which shows that, from the patient’s perspective, the quality of the dentist–patient relationship—reflected in clear explanations, active listening, respect, and patience—influences the overall evaluation of healthcare quality to a degree equal to or, in some cases, greater than technical competence itself [1,2,11]. This finding is further corroborated by a recent secondary analysis of dental patient satisfaction data, which identified the dentist domain—encompassing clinical competency, courtesy, and clarity of explanations—as the strongest predictor of overall patient satisfaction, outweighing structural factors such as appointment access or clinic environment [25]. It is particularly noteworthy that the attributes most frequently valued by patients—respect for privacy (88.2%), a calm and reassuring attitude (87.6%), attention to detail, and the absence of haste (86.8%)—predominantly relate to communication, interpersonal interaction, and professional attitude. Overall, communication and interpersonal skills were rated more highly than the other non-technical competency domains assessed in the present study, highlighting the particular importance patients assign to these aspects of dentist–patient interaction. By contrast, artistic skills, mainly related to dental aesthetics, received the lowest level of appreciation (49.9%). One possible explanation for the lower valuation of artistic skills is that these competencies may be perceived as particularly relevant to esthetic dental procedures, which may not be equally important to all patients. However, patients’ previous or anticipated use of esthetic dental procedures was not assessed in the present study; therefore, this interpretation remains speculative.

### 4.3. Demographic Differences

Women in the study consistently reported higher levels of agreement than men across all six categories of non-technical skills evaluated. The biggest difference was recorded for ethical and professional values (76.2% of women versus 60.4% of men). Female participants also placed significantly greater importance on receiving detailed explanations about their oral health status, available treatment options, and oral hygiene recommendations. This pattern is consistent with previous research showing that women generally attribute greater importance than men to communication, empathy, and relational aspects of healthcare [3]. From a practical perspective, these findings suggest that a communication style characterized by detailed explanations, comprehensive information, and proactive oral health counseling may be particularly valued by female patients. Nevertheless, such an approach should not be overlooked in the care of male patients, who, despite expressing slightly lower expectations, still placed a high value on communication skills, with 89.2% considering them important. This pattern is consistent with findings from a German study among adult dental patients, which similarly reported that women differed significantly from men in the importance assigned to dentists’ psycho-social competencies and personal presentation [3].

Comparative analysis across age groups revealed one of the most interesting patterns observed in the present study: younger patients reported higher expectations than older patients for nearly all categories of non-technical skills investigated. The differences were particularly pronounced for the dentist’s calm and reassuring attitude, respect for patient privacy, and willingness to listen patiently to patients’ concerns, with the importance assigned to these attributes declining progressively across increasing age groups. A plausible, though untested, explanation is that younger patients, having been exposed to increasingly high standards of service across multiple sectors—not only healthcare—may have developed higher expectations regarding the quality of professional interactions. Conversely, older generations may be more tolerant or less inclined to express dissatisfaction. However, these potential explanations were not directly investigated in the present study and should therefore be regarded as hypotheses rather than causal interpretations. It is noteworthy, however, that self-reported pain tolerance did not follow this linear pattern. The highest level was observed among patients aged 45–54 years, representing a small but interesting deviation from the otherwise consistent trend. This finding suggests a more complex, potentially non-linear relationship between age, cumulative experience with dental treatment, and the subjective perception of pain. Interestingly, this contrasts with findings from a German study among patients aged 35 years and older, which reported that the importance assigned to dentists’ psycho-social competencies increased, rather than decreased, with age [3]. This discrepancy may reflect differences in study populations—our sample spanned the full adult age range within a single Romanian city, whereas the German study focused on older adults across multiple cities—as well as potential cultural or healthcare-system differences between the two countries.

The most striking age-related difference, however, concerned the acceptance of digital technologies. The expectation that dentists should be familiar with telemedicine and the preference for communication through digital platforms declined markedly with increasing age, from over 60% among patients younger than 35 years to only 16–24% among those aged 65 years and older. This finding closely mirrors patterns consistently reported in the literature on digital health technology adoption, where perceived usefulness, digital self-efficacy, and privacy concerns have been shown to differentially influence younger and older adults’ willingness to use telemedicine services and online appointment platforms [5]. From a practical perspective, these findings suggest that the digitalization of dentist–patient communication—including appointment reminders, online scheduling, and remote pre-consultations—may represent a genuine competitive advantage for dental practices serving predominantly younger patients. Conversely, the same approach may be perceived by older adults as an additional barrier rather than a benefit, as they continue to favor more traditional, face-to-face interactions. Another noteworthy age-related finding was that the highest proportion of patients reporting that they could tolerate pain depending on the treatment was observed in the 45–54-year age group (55.5%). This pattern may plausibly reflect differences in previous dental experiences, treatment exposure, anxiety, or individual pain perception; however, these factors were not directly assessed in the present study. Therefore, this finding should be interpreted cautiously and warrants further investigation.

Patients residing in urban areas reported significantly higher levels of agreement than their rural counterparts for 6 of the 20 questionnaire items. This pattern was also observed across cognitive skills, communication skills, personal characteristics, and management competencies. These findings are in line with the international literature on rural–urban disparities in oral healthcare, which has documented both more limited access to high-quality dental services in rural settings and lower levels of patient satisfaction among rural populations [6]. One possible explanation is that rural patients, who are often faced with a more limited availability of dental services and consequently fewer opportunities for comparison, may calibrate their expectations at a lower level, a phenomenon that has also been reported in other areas of healthcare. An additional explanation relates to disparities in access to health information and differences in oral health literacy, which tends to be lower in rural populations [12]. Lower levels of oral health literacy may influence both patients’ ability to articulate specific expectations and their familiarity with contemporary standards of dentist–patient communication. We hypothesize that the lower expectations observed among rural patients may partly reflect more limited access to dental services and fewer opportunities for comparison between providers. Differences in oral health literacy may represent another potential explanatory factor. However, neither access-related experiences nor oral health literacy were directly assessed in the present study; therefore, these explanations remain speculative and should be interpreted with caution.

Patients with higher education (college or university degree) generally reported higher expectations than those with secondary education or no formal education. The largest differences were observed for cognitive skills, dentists’ personal characteristics, and the provision of detailed explanations regarding oral health status. This pattern is consistent with previous literature reporting associations between educational attainment and health literacy, including oral health literacy [6,12]. However, oral health literacy was not directly assessed in the present study and therefore cannot be inferred from educational level. It should be considered only as a possible explanatory variable for the observed differences, an interpretation that requires direct investigation in future studies. Interestingly, patients with no formal education assigned relatively greater importance to artistic skills than did the other educational groups. One possible, though untested, explanation is that this finding may reflect a more tangible, outcome-oriented perception of dental care, in which visible treatment outcomes are valued more highly than relational or cognitive dimensions. However, this interpretation was not directly assessed in the present study and should therefore be regarded as speculative.

A finding with important practical implications is that patients who attended regular dental check-ups (every six or twelve months) reported significantly higher expectations across four of the six categories of non-technical skills than those who sought dental care only in emergency situations. This interpretation is supported by a recent patient-based study conducted among older dental patients in Japan, which found that the adequacy of dentists’ explanations was significantly associated with regular attendance at dental check-ups, reinforcing the potential role of communication quality in sustaining preventive care behavior [26]. We propose two non-mutually exclusive hypotheses that may account for this finding; however, the cross-sectional design of the present study does not allow these potential explanations to be disentangled. On the one hand, patients who maintain a long-term and continuous relationship with their dentist may gradually develop clearer and more demanding expectations, potentially shaped by their accumulated experiences. On the other hand, it is equally plausible that a positive initial experience, particularly with regard to communication and the interpersonal relationship, may encourage patients to return for routine preventive care, whereas negative or superficial interactions may contribute to delaying subsequent visits and seeking dental care only when urgent treatment becomes necessary. These interpretations remain hypothetical, as the direction of the observed association cannot be established from the present cross-sectional data. Although the cross-sectional design of the present study does not allow the direction of this relationship to be established, these findings highlight the potential role of effective dentist–patient communication as a means of improving adherence to regular preventive dental check-ups. This is particularly relevant from a public health perspective, given that more than one-quarter of the participants (26.6%) reported visiting a dentist exclusively in emergency situations.

### 4.4. Clinical Implications

Beyond their descriptive value, the findings of the present study provide a basis for practical recommendations for the management of private dental practices, particularly within the local context of Iași. A previous pilot study conducted among patients attending private dental clinics in the same city reported that, although the majority of patients (74.3%) were satisfied with the clinical quality of the services received, a substantial proportion remained concerned about treatment costs. Moreover, patients’ willingness to recommend a dental practice to others was influenced more strongly by the dentist’s warmth and clarity of communication than by the technical equipment available in the practice [9]. The present findings expand on these observations by demonstrating that detailed explanations and an effective communication style are not only important determinants of overall patient satisfaction but are also valued differently across patient groups. This highlights the need for a tailored rather than a uniform approach to communication in dental practice.

A first area for intervention concerns staff education and professional training. Since communication and interpersonal skills were consistently ranked as the most important competencies by patients, regardless of age, sex, or place of residence, investing in the continuous training of both dentists and auxiliary staff in communication techniques—including active listening, the use of patient-friendly language, and the management of dental anxiety—appears to be one of the most effective strategies for improving the patient experience. Such training is expected to enhance not only immediate patient satisfaction but also adherence to regular dental check-ups and the development of long-term trust between patients and their dental care providers [1,10,11]. Such training initiatives should be grounded in structured, dentistry-specific models of patient-centered care; a recent narrative review proposed a theory-derived framework incorporating shared decision-making and a graded hierarchy of patient involvement, specifically adapted to the constraints of dental practice, which could inform the design of future training curricula [13]. This recommendation is particularly relevant in Romania, where differences in patient satisfaction between the public and private dental sectors appear to be attributable largely to the quality of communication and the efficiency of appointment scheduling, rather than solely to differences in technical resources or clinical equipment [10].

A second area for improvement concerns tailoring communication strategies to patients’ demographic characteristics. While the present findings establish an overall hierarchy of the importance of non-technical skills, they also demonstrate that there is no single communication approach that is equally effective for all patients. For younger, highly educated individuals, who showed greater interest in digital technologies, digital solutions—including online appointment scheduling, automated reminders, and telemedicine applications for preliminary consultations or patient triage—could be considered as options for responding to their reported preferences. However, the effectiveness of these approaches in improving patient-related outcomes was not assessed in the present study and requires prospective evaluation. For older adults and patients from rural areas, maintaining clear, accessible, and patient-centered communication may be particularly valuable. More generally, communication should be adapted to each patient’s individual needs and level of understanding, rather than assuming differences in oral health literacy based on demographic characteristics. Consequently, adopting a uniform communication strategy for all patients is unlikely to adequately address the different preferences and expectations observed across patient groups.

A third area of intervention relates to the role of communication in promoting adherence to preventive dental care. The finding that patients who attend regular dental check-ups reported higher expectations than those seeking care only in emergency situations indicates an association between dental attendance patterns and patients’ expectations regarding non-technical aspects of care. However, the direction of this association cannot be established from the present data, and no inference can be made regarding the quality of communication previously experienced by these patients. The mechanisms underlying this association warrant further investigation in longitudinal studies. From a public health perspective, this pattern is particularly important. Improving adherence to routine preventive care reduces both the long-term burden of oral disease and the complexity and cost of subsequent treatments. Effective communication appears to be one of the few determinants that dental practices can directly influence, unlike structural factors such as income, insurance coverage, or geographical accessibility, which largely remain beyond the control of individual practitioners [6,9].

### 4.5. Limitations and Future Directions of Research

The limitations of the present study should be acknowledged. First, its cross-sectional design does not allow causal relationships to be established between demographic characteristics and the perceptions investigated. For example, it cannot be determined with certainty whether the frequency of dental check-ups influences patients’ expectations or whether pre-existing expectations shape the frequency of dental attendance. Accordingly, the explanatory mechanisms proposed in the Discussion should be interpreted as hypotheses rather than as established causal relationships. Longitudinal studies or studies specifically designed to investigate these mechanisms are needed to determine the direction and nature of the observed associations.

Second, the study sample was drawn exclusively from private dental practices in a single Romanian city, which limits the generalizability of the findings to the broader Romanian population; the sample’s sociodemographic profile—predominantly female (59.3%), urban (69.4%), and with a comparatively high proportion of university-educated participants (40.7%)—does not mirror the sociodemographic structure of the general Romanian population, being consistent only with the profile of the patients who typically access private dental care. This specificity limits findings extrapolation to the Romanian patients who receive dental care within the public healthcare system, or to underrepresented groups such as rural and less-educated patients. In this context, we also faced a structural difficulty regarding the uneven distribution of patients across age groups—particularly the smaller subgroup of patients aged ≥65 years (n = 38)—which may have reduced the statistical power to detect smaller effect sizes within this subgroup, increasing the risk of Type II error, even though Fisher’s exact test was applied instead of the chi-square test to ensure the validity of the statistical comparisons.

In addition, the self-reported nature of the questionnaire introduces the possibility of social desirability bias. Furthermore, potential confounding factors that were not assessed in the present study may have contributed to the observed differences between sociodemographic groups. Oral health literacy was not directly assessed in the present study and should therefore be considered only as a possible explanatory variable rather than an established determinant of the observed differences. We also faced several methodological limitations as well: although patients were recruited consecutively within each participating practice, the possibility of selection bias cannot be excluded; the absence of multivariable adjustments for the associations identified through univariate comparisons does not allow to interpret them as independent effects, and residual confounding by unmeasured variables cannot be excluded.

Future multicenter studies involving both private and public dental settings, ideally complemented by direct assessments of patients’ oral health literacy and multivariable analysis techniques—using ordinal logistic regression or an alternative appropriate framework—would provide a more comprehensive understanding of whether the observed findings are consistent across populations with diverse socioeconomic backgrounds. Future studies should also extend beyond general dental practice to examine whether patients’ expectations regarding non-technical competencies differ across dental specialties (e.g., orthodontics, oral and maxillofacial surgery, pediatric dentistry, prosthodontics), since the nature of the doctor–patient interaction, treatment duration, and anxiety levels may vary substantially between these fields [8]. Likewise, direct comparative studies enrolling patients from both the private and public dental sectors within the same design, rather than separate analyses of each, would help clarify the extent to which patients’ expectations regarding soft skills are shaped by the healthcare delivery model itself [10]. Finally, although the questionnaire was specifically developed for patients within the DENTA_SOFT project by a multidisciplinary team including dental professionals and psychologists, further psychometric evaluation in independent and more diverse patient populations would strengthen the evidence regarding its validity, reliability, and generalizability.

## 5. Conclusions

The present study demonstrates that, from the patients’ perspective, communication and interpersonal skills were rated more highly than the other non-technical competency domains assessed. Respect, a calm and reassuring attitude, patience, and clear explanations regarding oral health emerged as the attributes most consistently valued by patients, regardless of their demographic characteristics.

Patients’ perceptions of the importance of non-technical skills varied significantly according to demographic characteristics. Women, younger patients, urban residents, individuals with higher educational attainment, and those attending regular dental check-ups consistently reported higher expectations across most categories of competencies assessed. The marked age-related differences observed in the acceptance of digital technologies and telemedicine further suggest that communication strategies may need to be responsive to the characteristics and preferences of different patient groups, rather than relying on a one-size-fits-all approach.

Overall, these findings support the implementation of personalized communication strategies in dental practice that take into account patients’ individual characteristics, preferences, and expectations. Such an approach may help better address the different perceptions and expectations regarding non-technical aspects of dental care identified across patient groups.

## Figures and Tables

**Table 1:** General demographic characteristics of the study patient cohort.

		n	%
Age Group	Up to 34 years	96	32.3
	35–44 years	58	19.5
	45–54 years	63	21.2
	55–64 years	42	14.1
	over 65 years	38	12.8
Sex	male	121	40.7
	female	176	59.3
Residence	urban	206	69.4
	rural	91	30.6
Educational level	High school (secondary education, completed with a baccalaureate diploma)	88	29.6
	College (post-secondary education comprising approximately 180 ECTS)	50	16.8
	University (university-level education comprising at least 240 ECTS)	121	40.7
	None of the above (educational attainment below high school/secondary level)	38	12.8
Frequency of dentist appointments	Every 6 months	59	19.9
	Once a year	98	33.0
	Less than once a year	61	20.5
	Only in emergencies	79	26.6
Total		297	100.0

**Table 2:** Detailed patient responses to the questionnaire.

	Patients’ Opinion									
	Agree to a Very Small Extent		Agree to a Small Extent		Agree to an Average Extent		Agree to a Large Extent		Agree to a Very Large Extent	
	n	%	n	%	n	%	n	%	n	%
Item 1	6	2.0	6	2.0	25	8.4	75	25.3	185	62.3
Item 2	4	1.3	9	3.0	22	7.4	85	28.6	177	59.6
Item 3	13	4.4	6	2.0	31	10.4	80	26.9	167	56.2
Item 4	7	2.4	17	5.7	23	7.7	77	25.9	173	58.2
Item 5	7	2.4	8	2.7	46	15.5	95	32.0	141	47.5
Item 6	36	12.1	85	28.6	61	20.5	55	18.5	60	20.2
Item 7	3	1.0	21	7.1	53	17.8	83	27.9	137	46.1
Item 8	4	1.3	20	6.7	65	21.9	96	32.3	112	37.7
Item 9	31	10.4	40	13.5	78	26.2	75	25.3	73	24.6
Item 10	27	9.1	38	12.8	56	18.9	79	26.6	97	32.7
Item 11	10	3.4	16	5.4	48	16.2	98	33.0	125	42.1
Item 12	3	1.0	10	3.4	33	11.1	86	29.0	165	55.6
Item 13	5	1.7	8	2.7	26	8.8	88	29.6	170	57.2
Item 14	6	2.0	10	3.4	59	19.9	98	33.0	124	41.8
Item 15	46	15.5	35	11.8	66	22.2	82	27.6	68	22.9
Item 16	73	24.6	45	15.2	65	21.9	49	16.5	65	21.9
Item 17	53	17.8	43	14.5	76	25.6	53	17.8	72	24.2
Item 18	21	7.1	40	13.5	83	27.9	77	25.9	76	25.6
Item 19	51	17.2	49	16.5	96	32.3	54	18.2	47	15.8
Item 20	63	21.2	59	19.9	83	27.9	47	15.8	45	15.2

## Data Availability

The data presented in this study are available on request from the corresponding author. The data are not publicly available due to privacy restrictions.

## Appendix A

The detailed questionnaire:

Please read the following statements carefully and indicate to what extent each one aligns with your opinion.

		**Agree to a Very Small Extent**	**Agree to a Small Extent**	**Agree to a Moderate extent**	**Agree to a Large Extent**	**Agree to a Very Large Extent**
1.	I would like my dentist to be a calm and confident person.	□	□	□	□	□
2.	I want my dentist to respect me and my privacy.	□	□	□	□	□
3.	I want my dentist to listen patiently to my health concerns.	□	□	□	□	□
4.	My dentist should provide me with detailed explanations about my dental condition and treatment options.	□	□	□	□	□
5.	It is very important to me that my dentist be punctual.	□	□	□	□	□
6.	Depending on the treatment, I can sometimes tolerate pain.	□	□	□	□	□
7.	It is important that my dentist use clear and simple language, both spoken and written.	□	□	□	□	□
8.	I would appreciate it if my dentist encouraged me in my efforts to improve my oral hygiene.	□	□	□	□	□
9.	I feel more comfortable when the dental office is decorated in pastel (warm) colors.	□	□	□	□	□
10.	I feel more comfortable when there is background music playing during dental procedures.	□	□	□	□	□
11.	I wish dental procedures wouldn’t take too long.	□	□	□	□	□
12.	I want the dental office staff to speak to me in a friendly and calm tone.	□	□	□	□	□
13.	I want the dentist to take his or her time during procedures and pay attention to detail.	□	□	□	□	□
14.	I appreciate it when my dentist gives me additional advice regarding my oral health habits.	□	□	□	□	□
15.	I want my dentist to be familiar with telemedicine (medical history, evaluation, and counseling in case of an emergency).	□	□	□	□	□
16.	If my dentist uses a laser instead of anesthesia, I’m no longer afraid.	□	□	□	□	□
17.	It’s much more efficient if my dentist contacts me through digital communication platforms to schedule appointments and provide long-term follow-up.	□	□	□	□	□
18.	Before treatment, it’s important to agree on a hand signal to use when I want a break.	□	□	□	□	□
19.	Except during treatment, I prefer that the dentist not wear a mask.	□	□	□	□	□
20.	I prefer to visit larger dental clinics rather than individual dental practices.	□	□	□	□	□

**Table A1:** Score classification thresholds for the six soft skills categories.

Soft Skills Categories	The Importance of Soft Skills in Work Performance				
	Very Low	Low	Moderate	High	Very High
Cognitive Skills	1–3	4–6	7–9	10–12	13–15
Communication and Interpersonal Skills	1–7	8–14	15–21	22–28	29–35
Artistic Skills	1	2	3	4	5
Ethical and Professional Values	1–5	6–10	11–15	16–20	21–25
Personal Characteristics	1, 2	3, 4	5, 6	7, 8	9, 10
Management Skills	1, 2	3, 4	5, 6	7, 8	9, 10

## Appendix B

**Table A2:** Detailed patient responses to the questionnaire—a comparative study by sexes.

	Agreement Extent	Sex				*p*-Value	Cramer’s V
		Male		Female			Effect Size
		N	%	N	%		
Item 1	very little	4	3.3%	2	1.1%	0.200	0.142
	little	4	3.3%	2	1.1%		(average)
	average	13	10.7%	12	6.8%		
	large	32	26.4%	43	24.4%		
	very large	68	56.2%	117	66.5%		
large + very large: %/CI 95%		82.6% 74.9% ÷ 88.4%		90.9% 85.7% ÷ 94.3%			
Item 2	very little	2	1.7%	2	1.1%	0.539	0.102
	little	5	4.1%	4	2.3%		(average)
	average	12	9.9%	10	5.7%		
	large	34	28.1%	51	29.0%		
	very large	68	56.2%	109	61.9%		
large + very large: %/CI 95%		84.3% 76.8% ÷ 89.7%		90.9% 85.7% ÷ 94.3%			
Item 3	very little	6	5.0%	7	4.0%	0.568	0.099
	little	4	3.3%	2	1.1%		(small)
	average	15	12.4%	16	9.1%		
	large	31	25.6%	49	27.8%		
	very large	65	53.7%	102	58.0%		
large + very large: %/CI 95%		79.3% 71.3% ÷ 85.6%		85.8% 79.9% ÷ 90.2%			
Item 4	very little	1	0.8%	6	3.4%	0.032	0.189
	little	10	8.3%	7	4.0%		(average)
	average	12	9.9%	11	6.3%		
	large	38	31.4%	39	22.2%		
	very large	60	49.6%	113	64.2%		
large + very large: %/CI 95%		81.0% 73.1% ÷ 87.0%		86.4% 80.5% ÷ 90.7%			
Item 5	very little	3	2.5%	4	2.3%	0.667	0.089
	little	5	4.1%	3	1.7%		(small)
	average	20	16.5%	26	14.8%		
	large	40	33.1%	55	31.3%		
	very large	53	43.8%	88	50.0%		
large + very large: %/CI 95%		76.9% 68.6% ÷ 83.5%		81.3% 74.8% ÷ 86.3%			
Item 6	very little	14	11.6%	22	12.5%	0.557	0.101
	little	37	30.6%	48	27.3%		(average)
	average	29	24.0%	32	18.2%		
	large	21	17.4%	34	19.3%		
	very large	20	16.5%	40	22.7%		
large + very large: %/CI 95%		33.9% 26.1% ÷ 42.7%		42.0% 35.0% ÷ 49.4%			
Item 7	very little	1	0.8%	2	1.1%	0.444	0.112
	little	10	8.3%	11	6.3%		(average)
	average	27	22.3%	26	14.8%		
	large	30	24.8%	53	30.1%		
	very large	53	43.8%	84	47.7%		
large + very large: %/CI 95%		68.6% 59.9% ÷ 76.2%		77.8% 71.1% ÷ 83.3%			
Item 8	very little	1	0.8%	3	1.7%	0.079	0.168
	little	10	8.3%	10	5.7%		(average)
	average	33	27.3%	32	18.2%		
	large	42	34.7%	54	30.7%		
	very large	35	28.9%	77	43.8%		
large + very large: %/CI 95%		63.6% 54.8% ÷ 71.7%		74.5% 67.5% ÷ 80.3%			
Item 9	very little	16	13.2%	15	8.5%	0.123	0.156
	little	20	16.5%	20	11.4%		(average)
	average	35	28.9%	43	24.4%		
	large	28	23.1%	47	26.7%		
	very large	22	18.2%	51	29.0%		
large + very large: %/CI 95%		41.3% 32.9% ÷ 50.2%		55.7% 48.3% ÷ 62.8%			
Item 10	very little	15	12.4%	12	6.8%	0.406	0.116
	little	17	14.0%	21	11.9%		(average)
	average	24	19.8%	32	18.2%		
	large	28	23.1%	51	29.0%		
	very large	37	30.6%	60	34.1%		
large + very large: %/CI 95%		53.7% 44.9% ÷ 62.4%		63.1% 55.7% ÷ 69.8%			
Item 11	very little	7	5.8%	3	1.7%	0.104	0.161
	little	7	5.8%	9	5.1%		(average)
	average	18	14.9%	30	17.0%		
	large	32	26.4%	66	37.5%		
	very large	57	47.1%	68	38.6%		
large + very large: %/CI 95%		73.5% 65.1% ÷ 80.6%		76.1% 69.3% ÷ 81.8%			
Item 12	very little	2	1.7%	1	0.6%	0.312	0.127
	little	5	4.1%	5	2.8%		(average)
	average	18	14.9%	15	8.5%		
	large	35	28.9%	51	29.0%		
	very large	61	50.4%	104	59.1%		
large + very large: %/CI 95%		79.3% 71.3% ÷ 85.6%		88.1% 82.4% ÷ 92.1%			
Item 13	very little	3	2.5%	2	1.1%	0.180	0.145
	little	5	4.1%	3	1.7%		(average)
	average	15	12.4%	11	6.3%		
	large	34	28.1%	54	30.7%		
	very large	64	52.9%	106	60.2%		
large + very large: %/CI 95%		81.0% 73.1% ÷ 87.0%		90.9% 85.7% ÷ 94.3%			
Item 14	very little	3	2.5%	3	1.7%	0.003	0.234
	little	5	4.1%	5	2.8%		(average)
	average	34	28.1%	25	14.2%		
	large	44	36.4%	54	30.7%		
	very large	35	28.9%	89	50.6%		
large + very large: %/CI 95%		65.3% 56.5% ÷ 73.2%		81.3% 74.8% ÷ 86.3%			
Item 15	very little	18	14.9%	28	15.9%	0.676	0.089
	little	18	14.9%	17	9.7%		(small)
	average	28	23.1%	38	21.6%		
	large	32	26.4%	50	28.4%		
	very large	25	20.7%	43	24.4%		
large + very large: %/CI 95%		47.1% 38.4% ÷ 56.0%		52.8% 45.5% ÷ 60.1%			
Item 16	very little	34	28.1%	39	22.2%	0.598	0.097
	little	20	16.5%	25	14.2%		(small)
	average	26	21.5%	39	22.2%		
	large	16	13.2%	33	18.8%		
	very large	25	20.7%	40	22.7%		
large + very large: %/CI 95%		33.9% 26.1% ÷ 42.7%		41.5% 34.5% ÷ 48.9%			
Item 17	very little	24	19.8%	29	16.5%	0.684	0.088
	little	18	14.9%	25	14.2%		(small)
	average	31	25.6%	45	25.6%		
	large	17	14.0%	36	20.5%		
	very large	31	25.6%	41	23.3%		
large + very large: %/CI 95%		39.6% 31.4% ÷ 48.6%		43.8% 36.6% ÷ 51.1%			
Item 18	very little	10	8.3%	11	6.3%	0.658	0.090
	little	17	14.0%	23	13.1%		(small)
	average	38	31.4%	45	25.6%		
	large	28	23.1%	49	27.8%		
	very large	28	23.1%	48	27.3%		
large + very large: %/CI 95%		46.2% 37.6% ÷ 55.1%		55.1% 47.7% ÷ 62.3%			
Item 19	very little	21	17.4%	30	17.0%	0.779	0.077
	little	24	19.8%	25	14.2%		(small)
	average	37	30.6%	59	33.5%		
	large	21	17.4%	33	18.8%		
	very large	18	14.9%	29	16.5%		
large + very large: %/CI 95%		32.3% 24.6% ÷ 41.0%		35.3% 28.6% ÷ 42.5%			
Item 20	very little	28	23.1%	35	19.9%	0.711	0.085
	little	20	16.5%	39	22.2%		(small)
	average	37	30.6%	46	26.1%		
	large	18	14.9%	29	16.5%		
	very large	18	14.9%	27	15.3%		
large + very large: %/CI 95%		29.8% 22.3% ÷ 38.4%		31.8% 25.4% ÷ 39.0%			
Total		121	100.0%	176	100.0%		

Pearson Chi-squared test.

**Table A3:** Patients’ summary opinions on the non-technical competencies that dentists should apply in practice—a comparative study by sexes.

The Importance of Soft Skills in Work Performance		Sex				*p*-Value Effect Size	Cramer’s V
		Male		Female			Effect Size
		N	%	N	%		
Cognitive Skills	very low	1	0.8%	2	1.1%	0.053	0.178
	low	2	1.7%	4	2.3%		(average)
	average	23	19.0%	15	8.5%		
	high	51	42.1%	68	38.6%		
	very high	44	36.4%	87	49.4%		
large + very large: %/CI 95%		78.5% 70.4% ÷ 84.9%		88.0% 82.4% ÷ 92.1%			
Communication and Interpersonal Skills	low	3	2.5%	3	1.7%	0.527	0.087
	average	10	8.3%	10	5.7%		(small)
	high	65	53.7%	87	49.4%		
	very high	43	35.5%	76	43.2%		
large + very large: %/CI 95%		89.2% 82.5% ÷ 93.6%		92.6% 87.8% ÷ 95.6%			
Artistic Skills	very low	16	13.2%	15	8.5%	0.123	0.156
	low	20	16.5%	20	11.4%		(average)
	average	35	28.9%	43	24.4%		
	high	28	23.1%	47	26.7%		
	very high	22	18.2%	51	29.0%		
large + very large: %/CI 95%		41.3% 32.9% ÷ 50.2%		55.7% 48.3% ÷ 62.8%			
Ethical and Professional Values	very low			1	0.6%	0.037	0.185
	low	3	2.5%	3	1.7%		(average)
	average	45	37.2%	38	21.6%		
	high	52	43.0%	89	50.6%		
	very high	21	17.4%	45	25.6%		
large + very large: %/CI 95%		60.4% 51.4% ÷ 68.6%		76.2% 69.3% ÷ 81.8%			
Personal Characteristics	very low	2	1.7%			0.084	0.166
	low	4	3.3%	3	1.7%		(average)
	average	25	20.7%	25	14.2%		
	high	43	35.5%	57	32.4%		
	very high	47	38.8%	91	51.7%		
large + very large: %/CI 95%		74.3% 65.9% ÷ 81.3%		84.1% 78.0% ÷ 88.8%			
Management Skills	very low	2	1.7%	3	1.7%	0.574	0.099
	low	8	6.6%	7	4.0%		(small)
	average	30	24.8%	35	19.9%		
	high	47	38.8%	69	39.2%		
	very high	34	28.1%	62	35.2%		
large + very large: %/CI 95%		66.9% 58.2% ÷ 74.7%		74.4% 67.5% ÷ 80.3%			
Total		121	100.0%	176	100.0%		

Pearson Chi-squared test.

**Table A4:** Detailed patient responses to the questionnaire—a comparative study by age group.

	Agreement Extent	Age Group										*p*-Value	Cramer’s V
		Up to 34 Years		35–44 Years		45–54 Years		55–64 Years		Over 65 Years			Effect Size
		N	%	N	%	N	%	N	%	N	%		
Item 1	very little	4	4.2%			2	3.2%					<0.001 *	0.202
	little			1	1.7%	1	1.6%	3	7.1%	1	2.6%		(average)
	average	4	4.2%	2	3.4%	6	9.5%	7	16.7%	6	15.8%		
	large	9	9.4%	18	31.0%	22	34.9%	15	35.7%	11	28.9%		
	very large	79	82.3%	37	63.8%	32	50.8%	17	40.5%	20	52.6%		
large + very large: %/CI 95%		91.7% 84.4% ÷ 95.7%		94.8% 85.9% ÷ 98.2%		85.7% 75.0% ÷ 92.3%		76.2% 61.5% ÷ 86.5%		81.5% 66.6% ÷ 90.8%			
Item 2	very little	3	3.1%			1	1.6%	0				0.002 *	0.179
	little	2	2.1%	1	1.7%	1	1.6%	3	7.1%	2	5.3%		(average)
	average	3	3.1%	3	5.2%	4	6.3%	7	16.7%	5	13.2%		
	large	15	15.6%	16	27.6%	24	38.1%	17	40.5%	13	34.2%		
	very large	73	76.0%	38	65.5%	33	52.4%	15	35.7%	18	47.4%		
large + very large: %/CI 95%		91.6% 84.4% ÷ 95.7%		93.1% 83.6% ÷ 97.3%		90.5% 80.7% ÷ 95.6%		76.2% 61.5% ÷ 86.5%		81.6% 66.6% ÷ 90.8%			
Item 3	very little	4	4.2%			5	7.9%	1	2.4%	3	7.9%	0.005	0.170
	little	1	1.0%					1	2.4%	4	10.5%		(average)
	average	6	6.3%	4	6.9%	9	14.3%	8	19.0%	4	10.5%		
	large	24	25.0%	16	27.6%	15	23.8%	14	33.3%	11	28.9%		
	very large	61	63.5%	38	65.5%	34	54.0%	18	42.9%	16	42.1%		
large + very large: %/CI 95%		88.5% 80.6% ÷ 93.5%		93.1% 83.6% ÷ 97.3%		77.8% 66.1% ÷ 86.3%		76.2% 61.5% ÷ 86.5%		71.0% 55.2% ÷ 83.0%			
Item 4	very little	3	3.1%			1	1.6%	1	2.4%	2	5.3%	0.078	0.144
	little	4	4.2%	1	1.7%	4	6.3%	4	9.5%	4	10.5%		(average)
	average	2	2.1%	8	13.8%	5	7.9%	5	11.9%	3	7.9%		
	large	18	18.8%	15	25.9%	19	30.2%	14	33.3%	11	28.9%		
	very large	69	71.9%	34	58.6%	34	54.0%	18	42.9%	18	47.4%		
large + very large: %/CI 95%		90.7% 83.1% ÷ 95.0%		84.5% 73.1% ÷ 91.6%		84.2% 73.2% ÷ 91.1%		76.2% 61.5% ÷ 86.5%		76.3% 60.8% ÷ 87.0%			
Item 5	very little	3	3.1%	1	1.7%	1	1.6%			2	5.3%	0.156	0.135
	little	2	2.1%	1	1.7%	1	1.6%	4	9.5%				(average)
	average	11	11.5%	6	10.3%	12	19.0%	9	21.4%	8	21.1%		
	large	27	28.1%	19	32.8%	20	31.7%	16	38.1%	13	34.2%		
	very large	53	55.2%	31	53.4%	29	46.0%	13	31.0%	15	39.5%		
large + very large: %/CI 95%		83.3% 74.6% ÷ 89.5%		86.2% 75.1% ÷ 92.8%		77.7% 66.1% ÷ 86.3%		69.1% 54.0% ÷ 80.9%		73.7% 58.0% ÷ 85.0%			
Item 6	very little	15	15.6%	8	13.8%	1	1.6%	6	14.3%	6	15.8%	0.018	0.159
	little	23	24.0%	23	39.7%	12	19.0%	16	38.1%	11	28.9%		(average)
	average	20	20.8%	10	17.2%	15	23.8%	9	21.4%	7	18.4%		
	large	22	22.9%	6	10.3%	12	19.0%	6	14.3%	9	23.7%		
	very large	16	16.7%	11	19.0%	23	36.5%	5	11.9%	5	13.2%		
large + very large: %/CI 95%		39.6% 30.4% ÷ 49.6%		29.3% 19.2% ÷ 42.0%		55.5% 43.3% ÷ 67.2%		26.2% 15.3% ÷ 41.1%		36.9% 23.4% ÷ 52.7%			
Item 7	very little	2	2.1%							1	2.6%	0.286	0.125
	little	5	5.2%	5	8.6%	6	9.5%	3	7.1%	2	5.3%		(average)
	average	15	15.6%	9	15.5%	11	17.5%	13	31.0%	5	13.2%		
	large	26	27.1%	18	31.0%	14	22.2%	16	38.1%	9	23.7%		
	very large	48	50.0%	26	44.8%	32	50.8%	10	23.8%	21	55.3%		
large + very large: %/CI 95%		77.1% 67.7% ÷ 84.4%		75.8% 63.5% ÷ 85.0%		73.0% 61.0% ÷ 82.4%		61.9% 46.8% ÷ 75.0%		79.0% 63.7% ÷ 88.9%			
Item 8	very little	3	3.1%							1	2.6%	0.047	0.149
	little	5	5.2%	4	6.9%	6	9.5%	2	4.8%	3	7.9%		(average)
	average	14	14.6%	11	19.0%	15	23.8%	17	40.5%	8	21.1%		
	large	31	32.3%	19	32.8%	15	23.8%	17	40.5%	14	36.8%		
	very large	43	44.8%	24	41.4%	27	42.9%	6	14.3%	12	31.6%		
large + very large: %/CI 95%		77.1% 67.7% ÷ 84.4%		74.2% 61.6% ÷ 83.7%		66.7% 54.4% ÷ 77.1%		54.8% 39.9% ÷ 68.8%		68.4% 52.5% ÷ 80.9%			
Item 9	very little	5	5.2%	4	6.9%	9	14.3%	6	14.3%	7	18.4%	0.044	0.150
	little	10	10.4%	6	10.3%	10	15.9%	5	11.9%	9	23.7%		(average)
	average	35	36.5%	9	15.5%	13	20.6%	13	31.0%	8	21.1%		
	large	23	24.0%	18	31.0%	14	22.2%	11	26.2%	9	23.7%		
	very large	23	24.0%	21	36.2%	17	27.0%	7	16.7%	5	13.2%		
large + very large: %/CI 95%		48.0% 38.2% ÷ 57.8%		67.2% 54.4% ÷ 77.9%		49.2% 37.3% ÷ 61.2%		42.9% 29.1% ÷ 57.8%		36.9% 23.4% ÷ 52.7%			
Item 10	very little	4	4.2%	2	3.4%	13	20.6%	5	11.9%	3	7.9%	<0.001 *	0.195
	little	6	6.3%	6	10.3%	7	11.1%	7	16.7%	12	31.6%		(average)
	average	22	22.9%	12	20.7%	5	7.9%	11	26.2%	6	15.8%		
	large	27	28.1%	12	20.7%	20	31.7%	12	28.6%	8	21.1%		
	very large	37	38.5%	26	44.8%	18	28.6%	7	16.7%	9	23.7%		
large + very large: %/CI 95%		66.6% 56.8% ÷ 75.3%		65.5% 52.7% ÷ 76.4%		60.3% 48.0% ÷ 71.5%		45.3% 31.2% ÷ 60.1%		44.8% 30.1% ÷ 60.3%			
Item 11	very little	2	2.1%			2	3.2%	4	9.5%	2	5.3%	0.041	0.151
	little	7	7.3%	4	6.9%			1	2.4%	4	10.5%		(average)
	average	13	13.5%	5	8.6%	15	23.8%	6	14.3%	9	23.7%		
	large	27	28.1%	20	34.5%	21	33.3%	17	40.5%	13	34.2%		
	very large	47	49.0%	29	50.0%	25	39.7%	14	33.3%	10	26.3%		
large + very large: %/CI 95%		77.1% 67.7% ÷ 84.4%		84.5% 73.1% ÷ 91.6%		73.0% 61.0% ÷ 82.4%		73.8% 58.9% ÷ 84.7%		60.5% 44.7% ÷ 74.4%			
Item 12	very little	2	2.1%			1	1.6%					0.142	0.136
	little	4	4.2%	1	1.7%	2	3.2%	1	2.4%	2	5.3%		(average)
	average	3	3.1%	6	10.3%	9	14.3%	10	23.8%	5	13.2%		
	large	24	25.0%	22	37.9%	16	25.4%	13	31.0%	11	28.9%		
	very large	63	65.6%	29	50.0%	35	55.6%	18	42.9%	20	52.6%		
large + very large: %/CI 95%		90.6% 83.1% ÷ 95.0%		87.9% 77.1% ÷ 94.0%		81.0% 69.6% ÷ 88.8%		73.9% 58.9% ÷ 84.7%		81.5% 66.6% ÷ 90.8%			
Item 13	very little	2	2.1%			1	1.6%			2	5.3%	0.143	0.136
	little	3	3.1%	2	3.4%	1	1.6%	1	2.4%	1	2.6%		(average)
	average	4	4.2%	4	6.9%	7	11.1%	8	19.0%	3	7.9%		
	large	21	21.9%	17	29.3%	20	31.7%	15	35.7%	15	39.5%		
	very large	66	68.8%	35	60.3%	34	54.0%	18	42.9%	17	44.7%		
large + very large: %/CI 95%		90.7% 83.1% ÷ 95.0%		89.6% 79.2% ÷ 95.2%		85.7% 75.0% ÷ 92.3%		78.6% 64.1% ÷ 88.3%		84.2% 69.6% ÷ 92.6%			
Item 14	very little	3	3.1%			1	1.6%			2	5.3%	0.037	0.152
	little	1	1.0%	1	1.7%	4	6.3%	1	2.4%	3	7.9%		(average)
	average	11	11.5%	11	19.0%	13	20.6%	14	33.3%	10	26.3%		
	large	28	29.2%	23	39.7%	21	33.3%	13	31.0%	13	34.2%		
	very large	53	55.2%	23	39.7%	24	38.1%	14	33.3%	10	26.3%		
large + very large: %/CI 95%		84.4% 75.8% ÷ 90.3%		79.4% 67.2% ÷ 87.7%		71.4% 59.3% ÷ 81.1%		64.3% 49.2% ÷ 77.0%		60.5% 44.7% ÷ 74.4%			
Item 15	very little	9	9.4%	4	6.9%	10	15.9%	11	26.2%	12	31.6%	0.008	0.166
	little	10	10.4%	8	13.8%	6	9.5%	6	14.3%	5	13.2%		(average)
	average	18	18.8%	18	31.0%	19	30.2%	6	14.3%	5	13.2%		
	large	26	27.1%	20	34.5%	16	25.4%	10	23.8%	10	26.3%		
	very large	33	34.4%	8	13.8%	12	19.0%	9	21.4%	6	15.8%		
large + very large: %/CI 95%		61.5% 51.5% ÷ 70.6%		48.3% 35.9% ÷ 60.8%		44.4% 32.8% ÷ 56.7%		45.2% 31.2% ÷ 60.1%		42.1% 27.9% ÷ 57.8%			
Item 16	very little	18	18.8%	14	24.1%	19	30.2%	13	31.0%	9	23.7%	0.644	0.106
	little	16	16.7%	9	15.5%	6	9.5%	6	14.3%	8	21.1%		(average)
	average	23	24.0%	16	27.6%	11	17.5%	7	16.7%	8	21.1%		
	large	15	15.6%	12	20.7%	9	14.3%	6	14.3%	7	18.4%		
	very large	24	25.0%	7	12.1%	18	28.6%	10	23.8%	6	15.8%		
large + very large: %/CI 95%		40.6% 31.3% ÷ 50.6%		32.8% 22.1% ÷ 45.6%		42.9% 31.4% ÷ 55.1%		38.1% 25.0% ÷ 53.2%		34.2% 21.2% ÷ 50.1%			
Item 17	very little	5	5.2%	7	12.1%	11	17.5%	14	33.3%	16	42.1%	<0.001 *	0.230
	little	9	9.4%	6	10.3%	12	19.0%	8	19.0%	8	21.1%		(average)
	average	23	24.0%	18	31.0%	17	27.0%	10	23.8%	8	21.1%		
	large	22	22.9%	18	31.0%	10	15.9%	2	4.8%	1	2.6%		
	very large	37	38.5%	9	15.5%	13	20.6%	8	19.0%	5	13.2%		
large + very large: %/CI 95%		61.4% 51.5% ÷ 70.6%		46.5% 34.3% ÷ 59.2%		36.5% 25.7% ÷ 48.9%		23.8% 13.5% ÷ 38.5%		15.8% 7.4% ÷ 30.4%			
Item 18	very little	3	3.1%	4	6.9%	7	11.1%	2	4.8%	5	13.2%	0.008 *	0.166
	little	8	8.3%	8	13.8%	8	12.7%	9	21.4%	7	18.4%		(average)
	average	24	25.0%	24	41.4%	17	27.0%	13	31.0%	5	13.2%		
	large	25	26.0%	17	29.3%	17	27.0%	6	14.3%	12	31.6%		
	very large	36	37.5%	5	8.6%	14	22.2%	12	28.6%	9	23.7%		
large + very large: %/CI 95%		63.5% 53.6% ÷ 72.5%		37.9% 26.6% ÷ 50.8%		49.2% 37.3% ÷ 61.2%		42.9% 29.1% ÷ 57.8%		55.3% 39.7% ÷ 69.9%			
Item 19	very little	15	15.6%	10	17.2%	13	20.6%	4	9.5%	9	23.7%	0.825	0.095
	little	13	13.5%	8	13.8%	9	14.3%	11	26.2%	8	21.1%		(small)
	average	34	35.4%	21	36.2%	17	27.0%	15	35.7%	9	23.7%		
	large	18	18.8%	11	19.0%	14	22.2%	5	11.9%	6	15.8%		
	very large	16	16.7%	8	13.8%	10	15.9%	7	16.7%	6	15.8%		
large + very large: %/CI 95%		35.5% 26.6% ÷ 45.4%		32.8% 22.1% ÷ 45.6%		38.1% 27.1% ÷ 50.4%		28.6% 17.2% ÷ 43.6%		31.6% 19.1% ÷ 47.5%			
Item 20	very little	16	16.7%	10	17.2%	15	23.8%	9	21.4%	13	34.2%	0.602	0.108
	little	18	18.8%	9	15.5%	12	19.0%	11	26.2%	9	23.7%		(average)
	average	28	29.2%	19	32.8%	16	25.4%	14	33.3%	6	15.8%		
	large	18	18.8%	12	20.7%	9	14.3%	3	7.1%	5	13.2%		
	very large	16	16.7%	8	13.8%	11	17.5%	5	11.9%	5	13.2%		
large + very large: %/CI 95%		35.5% 26.6% ÷ 45.4%		34.5% 23.6% ÷ 47.3%		31.8% 21.6% ÷ 44.0%		19.0% 10.0% ÷ 33.3%		26.4% 15.0% ÷ 42.0%			
Total		96	100.0%	58	100.0%	63	100.0%	42	100.0%	38	100.0%		

Pearson Chi-squared test; * *p* < 0.0025 statistically significant, according to the Bonferroni correction for multiple testing.

**Table A5:** Patients’ summary opinions on the non-technical competencies that dentists should use in practice—a comparative study by age group.

The Importance of Soft Skills in Work Performance		Age Group										*p*-Value	Cramer’s V
		Up to 34 Years		35–44 Years		45–54 Years		55–64 Years		Over 65 Years			Effect Size
		N	%	N	%	N	%	N	%	N	%		
Cognitive Skills	very low	1	1.0%							2	5.3%	<0.001 *	0.243
	low	2	2.1%			3	4.8%	1	2.4%				(average)
	average	5	5.2%	7	12.1%	4	6.3%	13	31.0%	9	23.7%		
	high	23	24.0%	22	37.9%	34	54.0%	20	47.6%	20	52.6%		
	very high	65	67.7%	29	50.0%	22	34.9%	8	19.0%	7	18.4%		
large + very large: %/CI 95%		91.7% 84.4% ÷ 95.7%		87.9% 77.1% ÷ 94.0%		88.9% 78.8% ÷ 94.5%		66.6% 51.6% ÷ 79.0%		71.0% 55.2% ÷ 83.0%			
Communication and Interpersonal Skills	low	3	3.1%			2	3.2%			1	2.6%	0.002 *	0.186
	average	2	2.1%	1	1.7%	5	7.9%	7	16.7%	5	13.2%		(average)
	high	38	39.6%	38	65.5%	33	52.4%	24	57.1%	19	50.0%		
	very high	53	55.2%	19	32.8%	23	36.5%	11	26.2%	13	34.2%		
large + very large: %/CI 95%		94.8% 88.4% ÷ 97.8%		98.3% 90.9% ÷ 99.7%		88.9% 78.8% ÷ 94.5%		83.3% 69.4% ÷ 91.7%		84.2% 69.6% ÷ 92.6%			
Artistic Skills	very low	5	5.2%	4	6.9%	9	14.3%	6	14.3%	7	18.4%	0.044	0.150
	low	10	10.4%	6	10.3%	10	15.9%	5	11.9%	9	23.7%		(average)
	average	35	36.5%	9	15.5%	13	20.6%	13	31.0%	8	21.1%		
	high	23	24.0%	18	31.0%	14	22.2%	11	26.2%	9	23.7%		
	very high	23	24.0%	21	36.2%	17	27.0%	7	16.7%	5	13.2%		
large + very large: %/CI 95%		48.0% 38.2% ÷ 57.8%		67.2% 54.4% ÷ 77.9%		49.2% 37.3% ÷ 61.2%		42.9% 29.1% ÷ 57.8%		36.9% 23.4% ÷ 52.7%			
Ethical and Professional Values	very low	1	1.0%									<0.001 *	0.188
	low	2	2.1%			1	1.6%			3	7.9%		(average)
	average	20	20.8%	10	17.2%	15	23.8%	22	52.4%	16	42.1%		
	high	46	47.9%	40	69.0%	30	47.6%	13	31.0%	12	31.6%		
	very high	27	28.1%	8	13.8%	17	27.0%	7	16.7%	7	18.4%		
large + very large: %/CI 95%		76.0% 66.6% ÷ 83.5%		82.8% 71.1% ÷ 90.4%		74.6% 62.7% ÷ 83.7%		47.7% 33.4% ÷ 62.3%		50.0% 34.8% ÷ 65.2%			
Personal Characteristics	very low					2	3.2%					<0.001*	0.214
	low	4	4.2%	1	1.7%			2	4.8%				(average)
	average	8	8.3%	2	3.4%	18	28.6%	10	23.8%	12	31.6%		
	high	26	27.1%	23	39.7%	16	25.4%	22	52.4%	13	34.2%		
	very high	58	60.4%	32	55.2%	27	42.9%	8	19.0%	13	34.2%		
large + very large: %/CI 95%		87.5% 79.4% ÷ 92.7%		94.9% 85.9% ÷ 98.2%		68.3% 56.0% ÷ 78.4%		71.4% 56.4% ÷ 82.8%		68.4% 52.5% ÷ 80.9%			
Management Skills	very low	2	2.1%			1	1.6%			2	5.3%	0.026	0.155
	low	3	3.1%	2	3.4%	3	4.8%	4	9.5%	3	7.9%		(average)
	average	15	15.6%	9	15.5%	16	25.4%	15	35.7%	10	26.3%		
	high	32	33.3%	29	50.0%	24	38.1%	14	33.3%	17	44.7%		
	very high	44	45.8%	18	31.0%	19	30.2%	9	21.4%	6	15.8%		
large + very large: %/CI 95%		79.1% 70.0% ÷ 86.1%		81.0% 69.1% ÷ 89.1%		68.3% 56.0% ÷ 78.4%		54.7% 39.9% ÷ 68.8%		60.5% 44.7% ÷ 74.4%			
Total		96	100.0%	58	100.0%	63	100.0%	42	100.0%	38	100.0%		

Pearson Chi-squared test; * *p* < 0.0083 statistically significant, according to the Bonferroni correction for multiple testing.

**Table A6:** Detailed patient responses to the questionnaire—comparative study by place of residence.

	Agreement Extent	Residence				*p*-Value	Cramer’s V
		Urban		Rural			Effect Size
		N	%	N	%		
Item 1	very little	4	1.9%	2	2.2%	0.011	0.210
	little	3	1.5%	3	3.3%		(average)
	average	11	5.3%	14	15.4%		
	large	48	23.3%	27	29.7%		
	very large	140	68.0%	45	49.5%		
large + very large: %/CI 95%		91.3% 86.6% ÷ 94.4%		79.2% 69.7% ÷ 86.2%			
Item 2	very little	3	1.5%	1	1.1%	<0.001 *	0.255
	little	2	1.0%	7	7.7%		(average)
	average	11	5.3%	11	12.1%		
	large	54	26.2%	31	34.1%		
	very large	136	66.0%	41	45.1%		
large + very large: %/CI 95%		92.2% 87.8% ÷ 95.2%		79.2% 69.7% ÷ 86.2%			
Item 3	very little	8	3.9%	5	5.5%	0.080	0.167
	little	2	1.0%	4	4.4%		(average)
	average	17	8.3%	14	15.4%		
	large	57	27.7%	23	25.3%		
	very large	122	59.2%	45	49.5%		
large + very large: %/CI 95%		86.9% 81.6% ÷ 90.8%		74.8% 64.9% ÷ 82.5%			
Item 4	very little	4	1.9%	3	3.3%	<0.001 *	0.339
	little	5	2.4%	12	13.2%		(large)
	average	8	3.9%	15	16.5%		
	large	52	25.2%	25	27.5%		
	very large	137	66.5%	36	39.6%		
large + very large: %/CI 95%		91.7% 87.2% ÷ 94.8%		67.1% 56.9% ÷ 75.8%			
Item 5	very little	7	3.4%			<0.001 *	0.273
	little	1	0.5%	7	7.7%		(average)
	average	25	12.1%	21	23.1%		
	large	68	33.0%	27	29.7%		
	very large	105	51.0%	36	39.6%		
large + very large: %/CI 95%		84.0% 78.4% ÷ 88.4%		69.3% 59.1% ÷ 77.8%			
Item 6	very little	29	14.1%	7	7.7%	0.010	0.211
	little	50	24.3%	35	38.5%		(average)
	average	37	18.0%	24	26.4%		
	large	42	20.4%	13	14.3%		
	very large	48	23.3%	12	13.2%		
large + very large: %/CI 95%		43.7% 37.1% ÷ 50.5%		27.5% 19.4% ÷ 37.4%			
Item 7	very little	3	1.5%			0.567	0.100
	little	15	7.3%	6	6.6%		(average)
	average	33	16.0%	20	22.0%		
	large	57	27.7%	26	28.6%		
	very large	98	47.6%	39	42.9%		
large + very large: %/CI 95%		75.3% 68.9% ÷ 80.6%		71.5% 61.4% ÷ 79.7%			
Item 8	very little	4	1.9%			0.007	0.218
	little	11	5.3%	9	9.9%		(average)
	average	36	17.5%	29	31.9%		
	large	67	32.5%	29	31.9%		
	very large	88	42.7%	24	26.4%		
large + very large: %/CI 95%		75.2% 68.9% ÷ 80.6%		58.3% 48.0% ÷ 67.8%			
Item 9	very little	16	7.8%	15	16.5%	0.026	0.193
	little	24	11.7%	16	17.6%		(average)
	average	56	27.2%	22	24.2%		
	large	51	24.8%	24	26.4%		
	very large	59	28.6%	14	15.4%		
large + very large: %/CI 95%		53.4% 46.6% ÷ 60.1%		41.8% 32.2% ÷ 52.0%			
Item 10	very little	12	5.8%	15	16.5%	0.030	0.190
	little	24	11.7%	14	15.4%		(average)
	average	39	18.9%	17	18.7%		
	large	59	28.6%	20	22.0%		
	very large	72	35.0%	25	27.5%		
large + very large: %/CI 95%		63.6% 56.8% ÷ 69.9%		49.5% 39.4% ÷ 59.5%			
Item 11	very little	6	2.9%	4	4.4%	0.469	0.110
	little	12	5.8%	4	4.4%		(average)
	average	29	14.1%	19	20.9%		
	large	67	32.5%	31	34.1%		
	very large	92	44.7%	33	36.3%		
large + very large: %/CI 95%		77.2% 71.0% ÷ 82.4%		70.4% 60.3% ÷ 78.7%			
Item 12	very little	2	1.0%	1	1.1%	0.039	0.184
	little	7	3.4%	3	3.3%		(average)
	average	15	7.3%	18	19.8%		
	large	63	30.6%	23	25.3%		
	very large	119	57.8%	46	50.5%		
large + very large: %/CI 95%		88.4% 83.3% ÷ 92.0%		75.8% 66.1% ÷ 83.5%			
Item 13	very little	4	1.9%	1	1.1%	0.001 *	0.248
	little	4	1.9%	4	4.4%		(average)
	average	9	4.4%	17	18.7%		
	large	65	31.6%	23	25.3%		
	very large	124	60.2%	46	50.5%		
large + very large: %/CI 95%		91.8% 87.2% ÷ 94.8%		75.8% 66.1% ÷ 83.5%			
Item 14	very little	4	1.9%	2	2.2%	<0.001 *	0.272
	little	4	1.9%	6	6.6%		(average)
	average	29	14.1%	30	33.0%		
	large	70	34.0%	28	30.8%		
	very large	99	48.1%	25	27.5%		
large + very large: %/CI 95%		82.1% 76.2% ÷ 86.7%		58.3% 48.0% ÷ 67.8%			
Item 15	very little	25	12.1%	21	23.1%	0.002 *	0.243
	little	18	8.7%	17	18.7%		(average)
	average	44	21.4%	22	24.2%		
	large	64	31.1%	18	19.8%		
	very large	55	26.7%	13	14.3%		
large + very large: %/CI 95%		57.8% 50.9% ÷ 64.3%		34.1% 25.2% ÷ 44.3%			
Item 16	very little	48	23.3%	25	27.5%	0.141	0.153
	little	29	14.1%	16	17.6%		(average)
	average	40	19.4%	25	27.5%		
	large	38	18.4%	11	12.1%		
	very large	51	24.8%	14	15.4%		
large + very large: %/CI 95%		43.2% 36.6% ÷ 50.0%		27.5% 19.4% ÷ 37.4%			
Item 17	very little	27	13.1%	26	28.6%	0.005	0.224
	little	31	15.0%	12	13.2%		(average)
	average	50	24.3%	26	28.6%		
	large	39	18.9%	14	15.4%		
	very large	59	28.6%	13	14.3%		
large + very large: %/CI 95%		47.5% 40.9% ÷ 54.4%		29.7% 21.3% ÷ 39.7%			
Item 18	very little	15	7.3%	6	6.6%	0.329	0.125
	little	23	11.2%	17	18.7%		(average)
	average	55	26.7%	28	30.8%		
	large	56	27.2%	21	23.1%		
	very large	57	27.7%	19	20.9%		
large + very large: %/CI 95%		54.9% 48.0% ÷ 61.5%		44.0% 34.2% ÷ 54.2%			
Item 19	very little	41	19.9%	10	11.0%	0.052	0.178
	little	27	13.1%	22	24.2%		(average)
	average	63	30.6%	33	36.3%		
	large	40	19.4%	14	15.4%		
	very large	35	17.0%	12	13.2%		
large + very large: %/CI 95%		36.4% 30.1% ÷ 43.2%		28.6% 20.3% ÷ 38.6%			
Item 20	very little	48	23.3%	15	16.5%	0.039	0.185
	little	41	19.9%	18	19.8%		(average)
	average	48	23.3%	35	38.5%		
	large	32	15.5%	15	16.5%		
	very large	37	18.0%	8	8.8%		
large + very large: %/CI 95%		33.5% 27.4% ÷ 40.2%		25.3% 17.5% ÷ 35.1%			
Total		206	100.0%	91	100.0%		

Pearson Chi-squared test; * *p* < 0.0025 statistically significant, according to the Bonferroni correction for multiple testing.

**Table A7:** Patients’ summary opinions on the non-technical competencies that dentists should apply in practice—a comparative study by place of residence.

The Importance of Soft Skills in Work Performance		Residence				*p*-Value	Cramer’s V
		Urban		Rural			Effect Size
		N	%	N	%		
Cognitive Skills	very low	2	1.0%	1	1.1%	<0.001 *	0.393
	low	4	1.9%	2	2.2%		(large)
	average	9	4.4%	29	31.9%		
	high	85	41.3%	34	37.4%		
	very high	106	51.5%	25	27.5%		
large + very large: %/CI 95%		92.8% 88.3% ÷ 95.5%		64.9% 54.6% ÷ 73.9%			
Communication and Interpersonal Skills	low	5	2.4%	1	1.1%	<0.001 *	0.252
	average	6	2.9%	14	15.4%		(average)
	high	103	50.0%	49	53.8%		
	very high	92	44.7%	27	29.7%		
large + very large: %/CI 95%		94.7% 90.7% ÷ 97.0%		83.5% 74.6% ÷ 89.7%			
Artistic Skills	very low	16	7.8%	15	16.5%	0.026	0.193
	low	24	11.7%	16	17.6%		(average)
	average	56	27.2%	22	24.2%		
	high	51	24.8%	24	26.4%		
	very high	59	28.6%	14	15.4%		
large + very large: %/CI 95%		53.4% 46.6% ÷ 60.1%		41.8% 32.2% ÷ 52.0%			
Ethical and Professional Values	very low	1	0.5%			0.012	0.209
	low	4	1.9%	2	2.2%		(average)
	average	45	21.8%	38	41.8%		
	high	106	51.5%	35	38.5%		
	very high	50	24.3%	16	17.6%		
large + very large: %/CI 95%		75.8% 69.4% ÷ 81.1%		56.1% 45.8% ÷ 65.8%			
Personal Characteristics	very low	1	0.5%	1	1.1%	<0.001 *	0.284
	low	5	2.4%	2	2.2%		(average)
	average	21	10.2%	29	31.9%		
	high	70	34.0%	30	33.0%		
	very high	109	52.9%	29	31.9%		
large + very large: %/CI 95%		86.9% 81.6% ÷ 90.8%		64.9% 54.6% ÷ 73.9%			
Management Skills	very low	5	2.4%			<0.001 *	0.330
	low	3	1.5%	12	13.2%		(large)
	average	35	17.0%	30	33.0%		
	high	89	43.2%	27	29.7%		
	very high	74	35.9%	22	24.2%		
large + very large: %/CI 95%		79.1% 73.1% ÷ 84.1%		53.9% 43.7% ÷ 63.7%			
Total		206	100.0%	91	100.0%		

Pearson Chi-squared test; * *p* < 0.0083 statistically significant, according to the Bonferroni correction for multiple testing.

**Table A8:** Detailed patient responses to the questionnaire—comparative study by educational level.

	Agreement Extent	Educational Level								*p*-Value	Cramer’s V
		High School		College		University		None of The Above			Effect Size
		N	%	N	%	N	%	N	%		
Item 1	very little	1	1.1%			5	4.1%			<0.001 *	0.210
	little	1	1.1%	2	4.0%			3	7.9%		(average)
	average	10	11.4%	3	6.0%	5	4.1%	7	18.4%		
	large	21	23.9%	20	40.0%	21	17.4%	13	34.2%		
	very large	55	62.5%	25	50.0%	90	74.4%	15	39.5%		
large + very large: %/CI 95%		86.4% 77.7% ÷ 92.0%		90.0% 78.6% ÷ 95.7%		91.8% 85.5% ÷ 95.4%		73.7% 58.0% ÷ 85.0%			
Item 2	very little	1	1.1%			3	2.5%			0.006 *	0.177
	little	2	2.3%	1	2.0%	2	1.7%	4	10.5%		(average)
	average	7	8.0%	3	6.0%	5	4.1%	7	18.4%		
	large	28	31.8%	16	32.0%	27	22.3%	14	36.8%		
	very large	50	56.8%	30	60.0%	84	69.4%	13	34.2%		
large + very large: %/CI 95%		88.6% 80.3% ÷ 93.7%		92.0% 81.2% ÷ 96.8%		91.7% 85.5% ÷ 95.4%		71.0% 55.2% ÷ 83.0%			
Item 3	very little	4	4.5%			7	5.8%	2	5.3%	0.142	0.139
	little	1	1.1%			2	1.7%	3	7.9%		(average)
	average	11	12.5%	8	16.0%	8	6.6%	4	10.5%		
	large	27	30.7%	15	30.0%	28	23.1%	10	26.3%		
	very large	45	51.1%	27	54.0%	76	62.8%	19	50.0%		
large + very large: %/CI 95%		81.8% 72.5% ÷ 88.5%		84.0% 71.5% ÷ 91.7%		85.9% 78.6% ÷ 91.0%		76.3% 60.8% ÷ 87.0%			
Item 4	very little	3	3.4%			3	2.5%	1	2.6%	0.032	0.159
	little	6	6.8%	2	4.0%	5	4.1%	4	10.5%		(average)
	average	11	12.5%	2	4.0%	3	2.5%	7	18.4%		
	large	21	23.9%	18	36.0%	30	24.8%	8	21.1%		
	very large	47	53.4%	28	56.0%	80	66.1%	18	47.4%		
large + very large: %/CI 95%		77.3% 67.5% ÷ 84.8%		92.0% 81.2% ÷ 96.8%		90.9% 84.5% ÷ 94.8%		68.5% 52.5% ÷ 80.9%			
Item 5	very little	1	1.1%			6	5.0%			0.266	0.128
	little	2	2.3%	1	2.0%	3	2.5%	2	5.3%		(average)
	average	15	17.0%	7	14.0%	14	11.6%	10	26.3%		
	large	26	29.5%	21	42.0%	37	30.6%	11	28.9%		
	very large	44	50.0%	21	42.0%	61	50.4%	15	39.5%		
large + very large: %/CI 95%		79.5% 70.0% ÷ 86.7%		84.0% 71.5% ÷ 91.7%		81.0% 73.1% ÷ 87.0%		68.4% 52.5% ÷ 80.9%			
Item 6	very little	8	9.1%	3	6.0%	19	15.7%	6	15.8%	0.047	0.154
	little	28	31.8%	17	34.0%	24	19.8%	16	42.1%		(average)
	average	20	22.7%	12	24.0%	20	16.5%	9	23.7%		
	large	18	20.5%	6	12.0%	28	23.1%	3	7.9%		
	very large	14	15.9%	12	24.0%	30	24.8%	4	10.5%		
large + very large: %/CI 95%		36.4% 27.1% ÷ 46.8%		36.0% 24.1% ÷ 49.9%		47.9% 39.2% ÷ 56.8%		18.4% 9.2% ÷ 33.4%			
Item 7	very little	1	1.1%			2	1.7%			0.093	0.145
	little	10	11.4%	4	8.0%	5	4.1%	2	5.3%		(average)
	average	12	13.6%	9	18.0%	18	14.9%	14	36.8%		
	large	24	27.3%	16	32.0%	32	26.4%	11	28.9%		
	very large	41	46.6%	21	42.0%	64	52.9%	11	28.9%		
large + very large: %/CI 95%		73.9% 63.8% ÷ 81.9%		74.0% 60.4% ÷ 84.1%		79.3% 71.3% ÷ 85.6%		57.8% 42.2% ÷ 72.1%			
Item 8	very little	1	1.1%			3	2.5%			0.003	0.183
	little	8	9.1%	3	6.0%	6	5.0%	3	7.9%		(average)
	average	20	22.7%	11	22.0%	15	12.4%	19	50.0%		
	large	29	33.0%	17	34.0%	41	33.9%	9	23.7%		
	very large	30	34.1%	19	38.0%	56	46.3%	7	18.4%		
large + very large: %/CI 95%		67.1% 56.7% ÷ 76.0%		72.0% 58.3% ÷ 82.5%		80.2% 72.2% ÷ 86.3%		42.1% 27.9% ÷ 57.8%			
Item 9	very little	16	18.2%	4	8.0%	8	6.6%	3	7.9%	0.026	0.161
	little	11	12.5%	9	18.0%	11	9.1%	9	23.7%		(average)
	average	23	26.1%	14	28.0%	36	29.8%	5	13.2%		
	large	18	20.5%	7	14.0%	37	30.6%	13	34.2%		
	very large	20	22.7%	16	32.0%	29	24.0%	8	21.1%		
large + very large: %/CI 95%		43.2% 33.3% ÷ 53.6%		46.0% 33.0% ÷ 59.6%		54.6% 45.7% ÷ 63.1%		55.3% 39.7% ÷ 69.9%			
Item 10	very little	11	12.5%	3	6.0%	8	6.6%	5	13.2%	0.317	0.124
	little	9	10.2%	10	20.0%	11	9.1%	8	21.1%		(average)
	average	19	21.6%	7	14.0%	24	19.8%	6	15.8%		
	large	22	25.0%	17	34.0%	32	26.4%	8	21.1%		
	very large	27	30.7%	13	26.0%	46	38.0%	11	28.9%		
large + very large: %/CI 95%		55.7% 45.3% ÷ 65.6%		60.0% 46.2% ÷ 72.4%		64.4% 55.6% ÷ 72.4%		50.0% 34.8% ÷ 65.2%			
Item 11	very little	4	4.5%	1	2.0%	4	3.3%	1	2.6%	0.861	0.088
	little	4	4.5%	4	8.0%	7	5.8%	1	2.6%		(small)
	average	18	20.5%	7	14.0%	17	14.0%	6	15.8%		
	large	24	27.3%	18	36.0%	39	32.2%	17	44.7%		
	very large	38	43.2%	20	40.0%	54	44.6%	13	34.2%		
large + very large: %/CI 95%		70.5% 60.2% ÷ 79.0%		76.0% 62.6% ÷ 85.7%		76.8% 68.6% ÷ 83.5%		78.9% 63.7% ÷ 88.9%			
Item 12	very little					3	2.5%			0.094	0.145
	little	2	2.3%	3	6.0%	5	4.1%				(average)
	average	10	11.4%	7	14.0%	7	5.8%	9	23.7%		
	large	26	29.5%	17	34.0%	32	26.4%	11	28.9%		
	very large	50	56.8%	23	46.0%	74	61.2%	18	47.4%		
large + very large: %/CI 95%		86.3% 77.7% ÷ 92.0%		80.0% 67.0% ÷ 88.8%		87.6% 80.6% ÷ 92.3%		76.3% 60.8% ÷ 87.0%			
Item 13	very little	1	1.1%			3	2.5%	1	2.6%	0.884	0.086
	little	2	2.3%	2	4.0%	3	2.5%	1	2.6%		(small)
	average	9	10.2%	6	12.0%	6	5.0%	5	13.2%		
	large	27	30.7%	14	28.0%	35	28.9%	12	31.6%		
	very large	49	55.7%	28	56.0%	74	61.2%	19	50.0%		
large + very large: %/CI 95%		86.4% 77.7% ÷ 92.0%		84.0% 71.5% ÷ 91.7%		90.1% 83.5% ÷ 94.2%		81.6% 66.6% ÷ 90.8%			
Item 14	very little	2	2.3%			3	2.5%	1	2.6%	0.215	0.132
	little	6	6.8%	1	2.0%	3	2.5%				(average)
	average	21	23.9%	13	26.0%	15	12.4%	10	26.3%		
	large	25	28.4%	16	32.0%	42	34.7%	15	39.5%		
	very large	34	38.6%	20	40.0%	58	47.9%	12	31.6%		
large + very large: %/CI 95%		67.0% 56.7% ÷ 76.0%		72.0% 58.3% ÷ 82.5%		82.6% 74.9% ÷ 88.4%		71.1% 55.2% ÷ 83.0%			
Item 15	very little	16	18.2%	5	10.0%	17	14.0%	8	21.1%	0.649	0.104
	little	13	14.8%	3	6.0%	13	10.7%	6	15.8%		(average)
	average	19	21.6%	16	32.0%	24	19.8%	7	18.4%		
	large	23	26.1%	15	30.0%	36	29.8%	8	21.1%		
	very large	17	19.3%	11	22.0%	31	25.6%	9	23.7%		
large + very large: %/CI 95%		45.4% 35.5% ÷ 55.8%		52.0% 38.5% ÷ 65.2%		55.4% 46.5% ÷ 63.9%		44.8% 30.1% ÷ 60.3%			
Item 16	very little	17	19.3%	17	34.0%	30	24.8%	9	23.7%	0.921	0.081
	little	13	14.8%	6	12.0%	19	15.7%	7	18.4%		(small)
	average	22	25.0%	8	16.0%	26	21.5%	9	23.7%		
	large	16	18.2%	9	18.0%	20	16.5%	4	10.5%		
	very large	20	22.7%	10	20.0%	26	21.5%	9	23.7%		
large + very large: %/CI 95%		40.9% 31.2% ÷ 51.4%		38.0% 25.9% ÷ 51.8%		38.0% 29.9% ÷ 46.9%		34.2% 21.2% ÷ 50.1%			
Item 17	very little	22	25.0%	8	16.0%	13	10.7%	10	26.3%	0.404	0.119
	little	10	11.4%	9	18.0%	18	14.9%	6	15.8%		(average)
	average	23	26.1%	13	26.0%	31	25.6%	9	23.7%		
	large	13	14.8%	11	22.0%	24	19.8%	5	13.2%		
	very large	20	22.7%	9	18.0%	35	28.9%	8	21.1%		
large + very large: %/CI 95%		37.5% 28.1% ÷ 47.9%		40.0% 27.6% ÷ 53.8%		48.7% 40.0% ÷ 57.6%		34.3% 21.2% ÷ 50.1%			
Item 18	very little	3	3.4%	5	10.0%	11	9.1%	2	5.3%	0.246	0.129
	little	11	12.5%	8	16.0%	12	9.9%	9	23.7%		(average)
	average	27	30.7%	16	32.0%	29	24.0%	11	28.9%		
	large	21	23.9%	15	30.0%	33	27.3%	8	21.1%		
	very large	26	29.5%	6	12.0%	36	29.8%	8	21.1%		
large + very large: %/CI 95%		53.4% 43.1% ÷ 63.5%		42.0% 29.4% ÷ 55.8%		57.1% 48.1% ÷ 65.5%		42.2% 27.9% ÷ 57.8%			
Item 19	very little	14	15.9%	9	18.0%	24	19.8%	4	10.5%	0.839	0.090
	little	17	19.3%	6	12.0%	17	14.0%	9	23.7%		(small)
	average	27	30.7%	18	36.0%	41	33.9%	10	26.3%		
	large	14	15.9%	11	22.0%	20	16.5%	9	23.7%		
	very large	16	18.2%	6	12.0%	19	15.7%	6	15.8%		
large + very large: %/CI 95%		34.1% 25.0% ÷ 44.5%		34.0% 22.4% ÷ 47.8%		32.2% 24.6% ÷ 41.0%		39.5% 25.6% ÷ 55.3%			
Item 20	very little	15	17.0%	13	26.0%	29	24.0%	6	15.8%	0.933	0.080
	little	20	22.7%	8	16.0%	25	20.7%	6	15.8%		(small)
	average	26	29.5%	15	30.0%	31	25.6%	11	28.9%		
	large	14	15.9%	7	14.0%	17	14.0%	9	23.7%		
	very large	13	14.8%	7	14.0%	19	15.7%	6	15.8%		
large + very large: %/CI 95%		30.7% 22.0% ÷ 41.0%		28.0% 17.5% ÷ 41.7%		29.7% 22.3% ÷ 38.4%		39.5% 25.6% ÷ 55.3%			
Total		88	100.0%	50	100.0%	121	100.0%	38	100.0%		

Pearson Chi-squared test; * *p* < 0.0025 statistically significant, according to the Bonferroni correction for multiple testing.

**Table A9:** Patients’ summary opinions on the cross-disciplinary competencies that dentists should apply in practice—a comparative study by educational level.

The Importance of Soft Skills in Work Performance		Educational Level								*p*-Value	Cramer’s V
		High School		College		University		None of The Above			Effect Size
		N	%	N	%	N	%	N	%		
Cognitive Skills	very low	1	1.1%			1	0.8%	1	2.6%	0.003 *	0.182
	low	2	2.3%			4	3.3%				(average)
	average	15	17.0%	7	14.0%	5	4.1%	11	28.9%		
	high	39	44.3%	24	48.0%	43	35.5%	13	34.2%		
	very high	31	35.2%	19	38.0%	68	56.2%	13	34.2%		
large + very large: %/CI 95%		79.5% 70.0% ÷ 86.7%		86.0% 73.8% ÷ 93.0%		91.7% 85.5% ÷ 95.4%		68.4% 52.5% ÷ 80.9%			
Communication and Interpersonal Skills	low	2	2.3%			4	3.3%			0.006 *	0.161
	average	7	8.0%	2	4.0%	5	4.1%	6	15.8%		(average)
	high	42	47.7%	34	68.0%	52	43.0%	24	63.2%		
	very high	37	42.0%	14	28.0%	60	49.6%	8	21.1%		
large + very large: %/CI 95%		89.7% 81.7% ÷ 94.5%		96.0% 86.5% ÷ 98.9%		92.6% 86.5% ÷ 96.0%		84.3% 69.6% ÷ 92.6%			
Artistic Skills	very low	16	18.2%	4	8.0%	8	6.6%	3	7.9%	0.026	0.161
	low	11	12.5%	9	18.0%	11	9.1%	9	23.7%		(average)
	average	23	26.1%	14	28.0%	36	29.8%	5	13.2%		
	high	18	20.5%	7	14.0%	37	30.6%	13	34.2%		
	very high	20	22.7%	16	32.0%	29	24.0%	8	21.1%		
large + very large: %/CI 95%		43.2% 33.3% ÷ 53.6%		46.0% 33.0% ÷ 59.6%		54.6% 45.7% ÷ 63.1%		55.3% 39.7% ÷ 69.9%			
Ethical and Professional Values	very low					1	0.8%			0.446	0.116
	low	2	2.3%			3	2.5%	1	2.6%		(average)
	average	25	28.4%	16	32.0%	25	20.7%	17	44.7%		
	high	42	47.7%	24	48.0%	63	52.1%	12	31.6%		
	very high	19	21.6%	10	20.0%	29	24.0%	8	21.1%		
large + very large: %/CI 95%		69.3% 59.0% ÷ 78.0%		68.0% 54.2% ÷ 79.2%		76.1% 67.7% ÷ 82.8%		52.7% 37.3% ÷ 67.5%			
Personal Characteristics	very low	1	1.1%			1	0.8%			0.022	0.163
	low	1	1.1%	1	2.0%	4	3.3%	1	2.6%		(average)
	average	16	18.2%	7	14.0%	13	10.7%	14	36.8%		
	high	31	35.2%	23	46.0%	34	28.1%	12	31.6%		
	very high	39	44.3%	19	38.0%	69	57.0%	11	28.9%		
large + very large: %/CI 95%		79.5% 70.0% ÷ 86.7%		84.0% 71.5% ÷ 91.7%		85.1% 77.7% ÷ 90.4%		60.5% 44.7% ÷ 74.4%			
Management Skills	very low	1	1.1%			4	3.3%			0.408	0.118
	low	5	5.7%	1	2.0%	5	4.1%	4	10.5%		(average)
	average	23	26.1%	10	20.0%	21	17.4%	11	28.9%		
	high	31	35.2%	24	48.0%	50	41.3%	11	28.9%		
	very high	28	31.8%	15	30.0%	41	33.9%	12	31.6%		
large + very large: %/CI 95%		67.0% 56.7% ÷ 76.0%		78.0% 64.8% ÷ 87.2%		75.2% 66.8% ÷ 82.0%		60.5% 44.7% ÷ 74.4%			
Total		88	100.0%	50	100.0%	121	100.0%	38	100.0%		

Pearson Chi-squared test; * *p* < 0.0083 statistically significant, according to the Bonferroni correction for multiple testing.

**Table A10:** Detailed patient responses to the questionnaire—comparative study based on the frequency of dental checkups.

	Agreement Extent	Frequency of Dentist Appointments								*p*-Value	Cramer’s V
		Every 6 Months		Once a Year		Less Than Once a Year		Only in Emergencies			Effect Size
		N	%	N	%	N	%	N	%		
Item 1	very little	3	5.1%	1	1.0%			2	2.5%	<0.001 *	0.204
	little	1	1.7%			1	1.6%	4	5.1%		(average)
	average	1	1.7%	3	3.1%	6	9.8%	15	19.0%		
	large	8	13.6%	31	31.6%	16	26.2%	20	25.3%		
	very large	46	78.0%	63	64.3%	38	62.3%	38	48.1%		
large + very large: %/CI 95%		91.6% 81.6% ÷ 96.3%		95.9% 90.0% ÷ 98.4%		88.5% 78.2% ÷ 94.3%		73.4% 62.8% ÷ 81.9%			
Item 2	very little	2	3.4%	1	1.0%			1	1.3%	0.004	0.180
	little	1	1.7%			3	4.9%	5	6.3%		(average)
	average	1	1.7%	3	3.1%	7	11.5%	11	13.9%		
	large	11	18.6%	30	30.6%	18	29.5%	26	32.9%		
	very large	44	74.6%	64	65.3%	33	54.1%	36	45.6%		
large + very large: %/CI 95%		93.2% 83.8% ÷ 97.3%		95.9% 90.0% ÷ 98.4%		83.6% 72.4% ÷ 90.8%		78.5% 68.2% ÷ 86.1%			
Item 3	very little	4	6.8%	3	3.1%			6	7.6%	0.038	0.157
	little	1	1.7%			2	3.3%	3	3.8%		(average)
	average	1	1.7%	8	8.2%	8	13.1%	14	17.7%		
	large	14	23.7%	30	30.6%	17	27.9%	19	24.1%		
	very large	39	66.1%	57	58.2%	34	55.7%	37	46.8%		
large + very large: %/CI 95%		89.8% 79.5% ÷ 95.3%		88.8% 81.0% ÷ 93.6%		83.6% 72.4% ÷ 90.8%		70.9% 60.1% ÷ 79.7%			
Item 4	very little	1	1.7%	2	2.0%	1	1.6%	3	3.8%	0.001 *	0.191
	little	2	3.4%	1	1.0%	5	8.2%	9	11.4%		(average)
	average			5	5.1%	6	9.8%	12	15.2%		
	large	10	16.9%	33	33.7%	17	27.9%	17	21.5%		
	very large	46	78.0%	57	58.2%	32	52.5%	38	48.1%		
large + very large: %/CI 95%		94.9% 86.1% ÷ 98.3%		91.9% 84.7% ÷ 95.8%		80.4% 68.7% ÷ 88.4%		69.6% 58.8% ÷ 78.7%			
Item 5	very little	3	5.1%	1	1.0%			3	3.8%	0.514	0.112
	little	1	1.7%	2	2.0%	3	4.9%	2	2.5%		(average)
	average	8	13.6%	12	12.2%	11	18.0%	15	19.0%		
	large	20	33.9%	36	36.7%	14	23.0%	25	31.6%		
	very large	27	45.8%	47	48.0%	33	54.1%	34	43.0%		
large + very large: %/CI 95%		79.7% 67.7% ÷ 88.0%		84.7% 76.3% ÷ 90.5%		77.1% 65.1% ÷ 85.8%		74.6% 64.1% ÷ 83.0%			
Item 6	very little	9	15.3%	9	9.2%	6	9.8%	12	15.2%	0.045	0.155
	little	11	18.6%	27	27.6%	27	44.3%	20	25.3%		(average)
	average	7	11.9%	22	22.4%	10	16.4%	22	27.8%		
	large	15	25.4%	20	20.4%	7	11.5%	13	16.5%		
	very large	17	28.8%	20	20.4%	11	18.0%	12	15.2%		
large + very large: %/CI 95%		54.2% 41.7% ÷ 66.3%		40.8% 31.6% ÷ 50.7%		29.5% 19.6% ÷ 41.9%		31.7% 22.4% ÷ 42.5%			
Item 7	very little	1	1.7%	1	1.0%			1	1.3%	0.322	0.124
	little	2	3.4%	7	7.1%	6	9.8%	6	7.6%		(average)
	average	5	8.5%	16	16.3%	13	21.3%	19	24.1%		
	large	14	23.7%	32	32.7%	17	27.9%	20	25.3%		
	very large	37	62.7%	42	42.9%	25	41.0%	33	41.8%		
large + very large: %/CI 95%		86.4% 75.5% ÷ 93.0%		75.6% 66.1% ÷ 83.0%		68.9% 56.4% ÷ 79.1%		67.1% 56.1% ÷ 76.4%			
Item 8	very little	2	3.4%	1	1.0%			1	1.3%	<0.001 *	0.234
	little	3	5.1%	6	6.1%	4	6.6%	7	8.9%		(average)
	average	2	3.4%	14	14.3%	23	37.7%	26	32.9%		
	large	13	22.0%	40	40.8%	19	31.1%	24	30.4%		
	very large	39	66.1%	37	37.8%	15	24.6%	21	26.6%		
large + very large: %/CI 95%		88.1% 77.5% ÷ 94.1%		78.6% 69.5% ÷ 85.5%		55.7% 43.3% ÷ 67.5%		57.0% 46.0% ÷ 67.3%			
Item 9	very little	3	5.1%	6	6.1%	7	11.5%	15	19.0%	0.023	0.163
	little	6	10.2%	8	8.2%	12	19.7%	14	17.7%		(average)
	average	19	32.2%	27	27.6%	14	23.0%	18	22.8%		
	large	13	22.0%	30	30.6%	11	18.0%	21	26.6%		
	very large	18	30.5%	27	27.6%	17	27.9%	11	13.9%		
large + very large: %/CI 95%		52.5% 40.0% ÷ 64.7%		58.2% 48.3% ÷ 67.4%		45.9% 34.0% ÷ 58.3%		40.5% 30.4% ÷ 51.5%			
Item 10	very little	2	3.4%	3	3.1%	11	18.0%	11	13.9%	<0.001 *	0.199
	little	4	6.8%	7	7.1%	13	21.3%	14	17.7%		(average)
	average	8	13.6%	21	21.4%	9	14.8%	18	22.8%		
	large	19	32.2%	34	34.7%	11	18.0%	15	19.0%		
	very large	26	44.1%	33	33.7%	17	27.9%	21	26.6%		
large + very large: %/CI 95%		76.3% 64.0% ÷ 85.3%		68.4% 58.6% ÷ 76.7%		45.9% 34.0% ÷ 58.3%		45.6% 35.0% ÷ 56.5%			
Item 11	very little	2	3.4%	2	2.0%	3	4.9%	3	3.8%	0.987	0.065
	little	4	6.8%	3	3.1%	3	4.9%	6	7.6%		(small)
	average	9	15.3%	16	16.3%	9	14.8%	14	17.7%		
	large	18	30.5%	35	35.7%	20	32.8%	25	31.6%		
	very large	26	44.1%	42	42.9%	26	42.6%	31	39.2%		
large + very large: %/CI 95%		74.6% 62.2% ÷ 83.9%		78.6% 69.5% ÷ 85.5%		75.4% 63.3% ÷ 84.5%		70.8% 60.1% ÷ 79.7%			
Item 12	very little	2	3.4%					1	1.3%	0.051	0.153
	little	1	1.7%	4	4.1%	2	3.3%	3	3.8%		(average)
	average	4	6.8%	8	8.2%	4	6.6%	17	21.5%		
	large	15	25.4%	26	26.5%	23	37.7%	22	27.8%		
	very large	37	62.7%	60	61.2%	32	52.5%	36	45.6%		
large + very large: %/CI 95%		88.1% 77.5% ÷ 94.1%		87.7% 79.8% ÷ 92.9%		90.2% 80.2% ÷ 95.4%		73.4% 62.8% ÷ 81.9%			
Item 13	very little	1	1.7%	1	1.0%			3	3.8%	0.141	0.139
	little	1	1.7%	1	1.0%	1	1.6%	5	6.3%		(average)
	average	3	5.1%	9	9.2%	5	8.2%	9	11.4%		
	large	11	18.6%	33	33.7%	20	32.8%	24	30.4%		
	very large	43	72.9%	54	55.1%	35	57.4%	38	48.1%		
large + very large: %/CI 95%		91.5% 81.6% ÷ 96.3%		88.8% 81.0% ÷ 93.6%		90.2% 80.2% ÷ 95.4%		78.5% 68.2% ÷ 86.1%			
Item 14	very little	2	3.4%	1	1.0%			3	3.8%	0.027	0.161
	little	1	1.7%	1	1.0%	3	4.9%	5	6.3%		(average)
	average	4	6.8%	19	19.4%	18	29.5%	18	22.8%		
	large	18	30.5%	35	35.7%	17	27.9%	28	35.4%		
	very large	34	57.6%	42	42.9%	23	37.7%	25	31.6%		
large + very large: %/CI 95%		88.1% 77.5% ÷ 94.1%		78.6% 69.5% ÷ 85.5%		65.6% 53.0% ÷ 76.3%		67.0% 56.1% ÷ 76.4%			
Item 15	very little	2	3.4%	13	13.3%	16	26.2%	15	19.0%	0.040	0.156
	little	5	8.5%	11	11.2%	9	14.8%	10	12.7%		(average)
	average	12	20.3%	24	24.5%	11	18.0%	19	24.1%		
	large	21	35.6%	27	27.6%	18	29.5%	16	20.3%		
	very large	19	32.2%	23	23.5%	7	11.5%	19	24.1%		
large + very large: %/CI 95%		67.8% 55.1% ÷ 78.3%		51.1% 41.3% ÷ 60.7%		41.0% 29.5% ÷ 53.5%		44.4% 33.9% ÷ 55.3%			
Item 16	very little	11	18.6%	24	24.5%	18	29.5%	20	25.3%	0.282	0.127
	little	6	10.2%	18	18.4%	14	23.0%	7	8.9%		(average)
	average	14	23.7%	19	19.4%	10	16.4%	22	27.8%		
	large	9	15.3%	17	17.3%	9	14.8%	14	17.7%		
	very large	19	32.2%	20	20.4%	10	16.4%	16	20.3%		
large + very large: %/CI 95%		47.5% 35.3% ÷ 60.0%		37.7% 28.8% ÷ 47.6%		31.2% 20.9% ÷ 43.6%		38.0% 28.1% ÷ 49.0%			
Item 17	very little	4	6.8%	10	10.2%	17	27.9%	22	27.8%	0.002 *	0.188
	little	5	8.5%	19	19.4%	11	18.0%	8	10.1%		(average)
	average	19	32.2%	21	21.4%	16	26.2%	20	25.3%		
	large	9	15.3%	23	23.5%	9	14.8%	12	15.2%		
	very large	22	37.3%	25	25.5%	8	13.1%	17	21.5%		
large + very large: %/CI 95%		52.6% 40.0% ÷ 64.7%		49.0% 39.3% ÷ 58.7%		27.9% 18.2% ÷ 40.2%		36.7% 26.9% ÷ 47.7%			
Item 18	very little	2	3.4%	6	6.1%	6	9.8%	7	8.9%	0.048	0.154
	little	6	10.2%	10	10.2%	14	23.0%	10	12.7%		(average)
	average	12	20.3%	27	27.6%	16	26.2%	28	35.4%		
	large	14	23.7%	30	30.6%	15	24.6%	18	22.8%		
	very large	25	42.4%	25	25.5%	10	16.4%	16	20.3%		
large + very large: %/CI 95%		66.1% 53.4% ÷ 76.9%		56.1% 46.3% ÷ 65.5%		41.0% 29.5% ÷ 53.5%		43.1% 32.7% ÷ 54.0%			
Item 19	very little	11	18.6%	15	15.3%	11	18.0%	14	17.7%	0.568	0.109
	little	3	5.1%	21	21.4%	11	18.0%	14	17.7%		(average)
	average	21	35.6%	29	29.6%	23	37.7%	23	29.1%		
	large	13	22.0%	19	19.4%	9	14.8%	13	16.5%		
	very large	11	18.6%	14	14.3%	7	11.5%	15	19.0%		
large + very large: %/CI 95%		40.6% 29.1% ÷ 53.4%		33.7% 25.1% ÷ 43.5%		26.3% 16.8% ÷ 38.4%		35.5% 25.8% ÷ 46.4%			
Item 20	very little	15	25.4%	16	16.3%	13	21.3%	19	24.1%	0.171	0.136
	little	6	10.2%	29	29.6%	14	23.0%	10	12.7%		(average)
	average	17	28.8%	21	21.4%	18	29.5%	27	34.2%		
	large	11	18.6%	15	15.3%	10	16.4%	11	13.9%		
	very large	10	16.9%	17	17.3%	6	9.8%	12	15.2%		
large + very large: %/CI 95%		35.5% 24.6% ÷ 48.3%		32.6% 24.2% ÷ 42.4%		26.2% 16.8% ÷ 38.4%		29.1% 20.3% ÷ 39.9%			
Total		59	100.0%	98	100.0%	61	100.0%	79	100.0%		

Pearson Chi-squared test; * *p* < 0.0025 statistically significant, according to the Bonferroni correction for multiple testing.

**Table A11:** Patients’ summary opinions on the non-technical competencies that dentists should use in practice—a comparative study based on the frequency of dental checkups.

The Importance of Soft Skills in Work Performance		Frequency of Dentist Appointments								*p*-Value	Cramer’s V
		Every 6 Months		Once a Year		Less Than Once a Year		Only in Emergencies			Effect Size
		N	%	N	%	N	%	N	%		
Cognitive Skills	very low			1	1.0%			2	2.5%	<0.001 *	0.214
	low	2	3.4%	1	1.0%	1	1.6%	2	2.5%		(average)
	average	2	3.4%	4	4.1%	14	23.0%	18	22.8%		
	high	16	27.1%	44	44.9%	25	41.0%	34	43.0%		
	very high	39	66.1%	48	49.0%	21	34.4%	23	29.1%		
large + very large: %/CI 95%		93.2% 83.8% ÷ 97.3%		93.9% 87.3% ÷ 97.2%		75.4% 63.3% ÷ 84.5%		72.1% 61.4% ÷ 80.8%			
Communication and Interpersonal Skills	low	2	3.4%	1	1.0%			3	3.8%	<0.001*	0.195
	average	1	1.7%	3	3.1%	6	9.8%	10	12.7%		(average)
	high	18	30.5%	52	53.1%	39	63.9%	43	54.4%		
	very high	38	64.4%	42	42.9%	16	26.2%	23	29.1%		
large + very large: %/CI 95%		94.9% 86.1% ÷ 98.3%		96.0% 90.0% ÷ 98.4%		90.1% 80.2% ÷ 95.4%		83.5% 73.9% ÷ 90.1%			
Artistic Skills	very low	3	5.1%	6	6.1%	7	11.5%	15	19.0%	0.023	0.163
	low	6	10.2%	8	8.2%	12	19.7%	14	17.7%		(average)
	average	19	32.2%	27	27.6%	14	23.0%	18	22.8%		
	high	13	22.0%	30	30.6%	11	18.0%	21	26.6%		
	very high	18	30.5%	27	27.6%	17	27.9%	11	13.9%		
large + very large: %/CI 95%		52.5% 40.0% ÷ 64.7%		58.2% 48.3% ÷ 67.4%		45.9% 34.0% ÷ 58.3%		40.5% 30.4% ÷ 51.5%			
Ethical and Professional Values	very low			1	1.0%					0.031	0.159
	low	2	3.4%			2	3.3%	2	2.5%		(average)
	average	8	13.6%	24	24.5%	25	41.0%	26	32.9%		
	high	28	47.5%	52	53.1%	23	37.7%	38	48.1%		
	very high	21	35.6%	21	21.4%	11	18.0%	13	16.5%		
large + very large: %/CI 95%		83.1% 71.5% ÷ 90.5%		74.5% 65.0% ÷ 82.1%		55.7% 43.3% ÷ 67.5%		64.6% 53.6% ÷ 74.2%			
Personal Characteristics	very low							2	2.5%	<0.001 *	0.235
	low	3	5.1%	1	1.0%	1	1.6%	2	2.5%		(average)
	average	2	3.4%	7	7.1%	16	26.2%	25	31.6%		
	high	14	23.7%	37	37.8%	23	37.7%	26	32.9%		
	very high	40	67.8%	53	54.1%	21	34.4%	24	30.4%		
large + very large: %/CI 95%		91.5% 81.6% ÷ 96.3%		91.9% 84.7% ÷ 95.8%		72.1% 59.8% ÷ 81.8%		63.3% 52.3% ÷ 73.1%			
Management Skills	very low	1	1.7%	1	1.0%			3	3.8%	0.005 *	0.179
	low	2	3.4%	1	1.0%	9	14.8%	3	3.8%		(average)
	average	6	10.2%	22	22.4%	14	23.0%	23	29.1%		
	high	26	44.1%	44	44.9%	19	31.1%	27	34.2%		
	very high	24	40.7%	30	30.6%	19	31.1%	23	29.1%		
large + very large: %/CI 95%		84.8% 73.5% ÷ 91.8%		75.5% 66.1% ÷ 83.0%		62.2% 49.7% ÷ 73.4%		63.3% 52.3% ÷ 73.1%			
Total		59	100.0%	98	100.0%	61	100.0%	79	100.0%		

Pearson Chi-squared test; * *p* < 0.0083 statistically significant, according to the Bonferroni correction for multiple testing.
